# Partial correction of immunodeficiency by lentiviral vector gene therapy in mouse models carrying *Rag1* hypomorphic mutations

**DOI:** 10.3389/fimmu.2023.1268620

**Published:** 2023-11-13

**Authors:** Maria Carmina Castiello, Martina Di Verniere, Elena Draghici, Elena Fontana, Sara Penna, Lucia Sereni, Alessandra Zecchillo, Denise Minuta, Paolo Uva, Marco Zahn, Irene Gil-Farina, Andrea Annoni, Silvia Iaia, Lisa M. Ott de Bruin, Luigi D. Notarangelo, Karin Pike-Overzet, Frank J. T. Staal, Anna Villa, Valentina Capo

**Affiliations:** ^1^San Raffaele-Telethon Institute for Gene Therapy, IRCCS San Raffaele Scientific Institute, Milan, Italy; ^2^Milan Unit, Istituto di Ricerca Genetica e Biomedica, Consiglio Nazionale delle Ricerche, Milan, Italy; ^3^Humanitas Clinical and Research Center, IRCCS, Rozzano, Milan, Italy; ^4^Clinical Bioinformatics, IRCCS Istituto Giannina Gaslini, Genoa, Italy; ^5^ProtaGene CGT GmbH, Heidelberg, Germany; ^6^Willem-Alexander Children’s Hospital, Department of Pediatrics, Pediatric Stem Cell Transplantation Program, Leiden University Medical Center, Leiden, Netherlands; ^7^Department of Immunology, Leiden University Medical Center, Leiden, Netherlands; ^8^Laboratory of Clinical Immunology and Microbiology, National Institute of Allergy and Infectious Diseases (NIAID), National Institutes of Health (NIH), Bethesda, MD, United States

**Keywords:** *RAG1* gene, gene therapy, immunodeficiency, immune dysregulation, lentiviral vectors, autoimmunity, leaky SCID

## Abstract

**Introduction:**

Recombination activating genes (*RAG*) 1 and 2 defects are the most frequent form of severe combined immunodeficiency (SCID). Patients with residual RAG activity have a spectrum of clinical manifestations ranging from Omenn syndrome to delayed-onset combined immunodeficiency, often associated with granulomas and/or autoimmunity (CID-G/AI). Lentiviral vector (LV) gene therapy (GT) has been proposed as an alternative treatment to the standard hematopoietic stem cell transplant and a clinical trial for RAG1 SCID patients recently started. However, GT in patients with hypomorphic RAG mutations poses additional risks, because of the residual endogenous RAG1 expression and the general state of immune dysregulation and associated inflammation.

**Methods:**

In this study, we assessed the efficacy of GT in 2 hypomorphic Rag1 murine models (Rag1^F971L/F971L^ and Rag1^R972Q/R972Q^), exploiting the same LV used in the clinical trial encoding RAG1 under control of the MND promoter.

**Results and discussion:**

Starting 6 weeks after transplant, GT-treated mice showed a decrease in proportion of myeloid cells and a concomitant increase of B, T and total white blood cells. However, counts remained lower than in mice transplanted with WT Lin- cells. At euthanasia, we observed a general redistribution of immune subsets in tissues, with the appearance of mature recirculating B cells in the bone marrow. In the thymus, we demonstrated correction of the block at double negative stage, with a modest improvement in the cortical/medullary ratio. Analysis of antigenspecific IgM and IgG serum levels after *in vivo* challenge showed an amelioration of antibody responses, suggesting that the partial immune correction could confer a clinical benefit. Notably, no overt signs of autoimmunity were detected, with B-cell activating factor decreasing to normal levels and autoantibodies remaining stable after GT. On the other hand, thymic enlargement was frequently observed, although not due to vector integration and insertional mutagenesis. In conclusion, our work shows that GT could partially alleviate the combined immunodeficiency of hypomorphic RAG1 patients and that extensive efficacy and safety studies with alternative models are required before commencing RAG gene therapy in thesehighly complex patients.

## Introduction

Recombinase-activating gene (*RAG*) 1 and 2 are responsible for the rearrangement of variable (V), diversity (D) and joining (J) coding elements of the Immunoglobulin (Ig) and T cell receptor (TCR) genes. This process is fundamental to generate a diverse antigen-specific Ig and TCR repertoire necessary to properly respond to pathogens and ensure immune tolerance (1). Genetic defects that abrogate RAG1 or RAG2 function lead to the T- B- severe combined immunodeficiency (SCID), a life-threatening disorder of the adaptive immune system. SCID patients suffer from recurrent infections and require hematopoietic stem cell transplantation (HSCT) to survive through childhood (2). On the other hand, hypomorphic mutations inducing residual RAG function cause a spectrum of manifestations, ranging from Omenn syndrome to leaky SCID and to combined immune deficiency associated with granulomas and/or autoimmunity (CID-G/AI). These forms result in the generation of few oligoclonal T cells (and often also B cells, in the case of CID-G/AI) with molecular signatures of self-reactivity, causing a combination of severe immunodeficiency and inflammation or autoimmunity, and also require HSCT as a curative treatment (3).

Although HSCT represents the standard of care for *RAG* deficiencies, it is still associated with high risk of complications for patients affected by the leakiest forms (CID-G/AI) (4–6) and even for full blown SCID patients, allogeneic procedures still are associated with significant mortality of around 25% (7). Lentiviral vector (LV) gene therapy (GT) has been proposed as an alternative treatment and different constructs with different promoters have been tested, especially in *Rag1* or *Rag2* knock out mice that recapitulate the T- B- SCID phenotype (8–13). The LV carrying the codon-optimized (c.o.) *RAG1* therapeutic gene under the control of the *MND* (myeloproliferative sarcoma virus enhancer, negative control region deleted, dl587rev primer binding site substituted) promoter allowed the highest level of *RAG1* expression in correlation to vector copy number/genome (VCN), and achieved the best immune reconstitution in *Rag1^-/-^* mice (10).

Recently, the first 2 *RAG1*-SCID patients were treated in the context of a phase I/II clinical trial with this LV (ClinicalTrials.gov Identifier: NCT04797260 (10, 14),).

The current clinical trial excludes patients with hypomorphic mutations, due to the unknown risks caused by the complex setting of immune dysregulation and residual T cells (10). To assess the efficacy and safety of GT in this context, we tested the MND-c.o.RAG1 LV in two different mouse models carrying hypomorphic *Rag1* mutations, the *Rag1^F971L/F971L^* and the *Rag1^R972Q/R972Q^* mice. These mutations are equivalent to those found in patients with CID and recapitulate many aspects of the human phenotype, such as the presence of few T and B cells, skewed B and T cell receptor repertoires and production of autoantibodies (15). Mutant mice offer an important tool to evaluate the impact of residual autoreactive lymphocytes after GT and how they may affect the selective advantage that favors GT efficacy in the *Rag1^-/-^* model. In this work, we transplanted these mouse models with transduced GT cells and evaluated the immune reconstitution, while monitoring the onset of autoimmunity and adverse events. Only partial correction of the B and T cell counts was achieved in GT-treated mice, that however were able to mount antigen-specific immunoglobulins. No overt signs of autoimmunity were detected, but increased rate of thymic hyperplasia was observed, apparently unrelated to vector integrations.

## Materials and methods

### Mice

Animal experimental procedures were approved by the Institutional Animal Care and Use Committee (IACUC) of San Raffaele Hospital and Italian Ministry of Health. C57Bl/6 wild-type (WT) mice were purchased from Charles River Laboratories (USA). The *Rag1^F971L/F971L^* and the *Rag1^R972Q/R972Q^* mice were previously described (15) and were bred and maintained in the Ospedale San Raffaele (OSR) animal facility (IACUC n. 877, project prot.6EEAF.48, ministry authorization n. 113/2018-PR and IACUC n. 1125, project prot.6EEAF.164, ministry authorization n.5/2021-PR).

### Transduction of murine lineage-negative cells

Femurs and tibiae of 6- to 12-week-old donor mice were flushed and passed through a 0.4-μm cell strainer. Lineage-negative (Lin-) cells were obtained using the Lineage Cell Depletion kit (Miltenyi Biotec), following manufacturer’s instructions. Lin- cells were transduced as previously described (10) with the pre-clinical GMP batch of the MND-c.o.RAG1 vector. Untransduced Lin- cells from mutant and WT mice were cultured in parallel.

After transduction, cells were divided for *in vivo* transplantation, liquid culture for VCN determination and colony forming unit (CFU) assay. For *in vitro* liquid culture, cells were kept in culture in stimulation medium (10) for 10 days and then collected to extract DNA.

For CFU assay, cells were plated in MethoCult GF M3434 (StemCell Technologies) medium at a density of 1000 cells/ml and cultured for 10-12 days. Single cell colonies were counted and picked for VCN determination. In addition, remaining bulk cultures were collected for DNA extraction and VCN determination.

### Transplantation of *Rag1* mutant mice

Six- to 10-week-old recipient mice were conditioned with lethal total-body irradiation (TBI, 8 Gy, split dose) at least 2 hours before transplantation and were then injected in the caudal vein with 0.5-1x10^6^ Lin- cells. GT mice received transduced *Rag1^F971L/F971L^* or *Rag1^R972Q/R972Q^* Lin- cells, while BMT WT and BMT UT mice were transplanted with untransduced WT or mutant *Rag1* cells, respectively. Gentamicin sulfate (Italfarmaco, Milan, Italy) was administered in drinking water (8 μg/mL) for the first 2 weeks after transplantation to prevent infections.

### DNA extraction and vector copy number determination

DNA extraction from liquid cultures, tissue single cell suspensions and bulk CFU cultures was performed with QIAamp DNA micro or mini kits (QIAGEN), following manufacturer’s instructions. DNA extraction from single CFU colonies was performed using QuickExtract DNA extraction Solution, following manufacturer’s instructions. Vector copy number was quantified by digital droplet PCR as previously described (13).

### Flow cytometry

Single-cell suspensions from tissues were obtained smashing tissues through a 0.4-μm cell strainer. Red blood cells of PB were lysed to obtain WBC. Single-cell suspensions and WBC were stained for 15 min at room temperature with the following antibodies from BD Pharmingen, Miltenyi Biotec, BioLegend or eBioscience: CD3, CD4, CD8, CD11b, CD19, CD21, CD23, CD24, CD25, CD43, CD44, CD45.1, CD45.2, CD48, CD62 ligand, CD69, CD117, CD150, B220, IgM, IgD, NKp46, NK1.1, Lin+ cocktail, and Sca1. Viability was determined by using the Live/Dead Fixable Dead Cell Stain Kit (Thermo Fisher Scientific). Samples were acquired on a FACSCanto II (BD Biosciences) and analyzed with FlowJo software.

### Immunization

Four months after GT, mice were injected intravenously with 100 μg of TNP-KLH (Biosearch Technologies), to elicit a T-dependent response. Three weeks later, animals were injected intraperitoneally (i.p.) with 100 μg of TNP-KLH to boost the immune response. Serum was collected weekly. Antigen-specific IgM and IgG antibodies were evaluated in serum by ELISA, as previously described (13).

Alternatively, mice were challenged with 50 μg of TNP-Ficoll (Biosearch Technologies) by i.p. injection and serum was collected after 2 weeks. Antigen-specific IgG3 antibodies were evaluated in serum by ELISA, as previously described (15).

### Determination of VCN

Genomic DNA was extracted with the QIAamp DNA Blood Mini Kit (Qiagen, Hilden, Germany), according to the manufacturer’s instructions. Vector copy number/genome (VCN) was quantified on 10 ng of genomic DNA using the QX200 Droplet Digital PCR System (Bio-Rad Laboratories), as previously described (13).

### Serum immunoglobulin, BAFF and autoantibody quantification

Total IgA, IgG, and IgM levels were quantified on serum or plasma samples obtained at termination by using the MILLIPLEX MAP Mouse Immunoglobulin Isotyping Magnetic Bead Panel and MAGPIX System (Merck Millipore), according to the manufacturer’s instructions.

B cell–activating factor (BAFF) levels were analyzed with the mouse BAFF Quantikine ELISA (R&D Systems), according to the manufacturer’s instructions.

Protein arrays were used to screen for a broad panel of IgG autoantibodies (University of Texas Southwestern Medical Center, Genomic and Microarray Core Facility). Serum obtained 6 or 12 months after transplant was used. The raw signals were background subtracted and normalized to generate Normalized Signal Intensities (NSI). The matrix of NSI data was used to create heatmaps, where each value represents the fold-change on the log2 scale, using the row-wise average of BMT WT as a reference.

### TCR repertoire analysis

Total RNA was obtained from spleen cells with the RNeasy Mini Kit (Qiagen), according to the manufacturer’s instructions. TCR beta chain clonotypes were identified by RACE-PCR (rapid amplification of cDNA ends-PCR) followed by deep sequencing (ProtaGene). Briefly, between 6.6ng and 720ng RNA input were used for cDNA synthesis using a TCR constant region specific primer followed by the addition of an adaptor oligo molecule at the 5’cDNA end. cDNA was AMPure XP purified and applied to two subsequent nested PCR reactions using primers binding to the beta chain constant region and the introduced adaptor. In the second amplification step, fusion primers enabling sequencing on Illumina MiSeq platform were used and PCR products were purified using AMPure XP beads. Finally, products were pooled and sequenced in asymmetric 400bp+100bp paired-end mode.

Sequencing data were analyzed using MiGEC tool (https://migec.readthedocs.io/en/latest) in order to sort the sequences by the sample specific barcodes introduced during library preparation followed by clustering based on unique molecular identifiers. Sorted sequences were then analyzed with MiXCR (https://mixcr.readthedocs.io/en/master/index.html) for the identification of the TCR beta clonotypes and their respective relative frequencies. The top 10 clonotype frequencies were taken into account to receive an estimation on the clonality and the TCR beta repertoire of the samples. Further downstream analyses were performed using VDJtools (https://vdjtools-doc.readthedocs.io/en/master/) that allowed the evaluation of V- and J-gene usage of the single clonotypes and the display of the gene recombination, as well as CDR3 sequence length distribution.

### Integration site analysis

Standard S-EPTS/LM-PCR (shearing extension primer tag selection ligation-mediated PCR) and deep sequencing were performed to identify lentiviral vector flanking genomic sequences (ProtaGene) (16–18). Inputs between 16ng and 400ng of genomic DNA were used.

Briefly, input genomic DNA was sheared to a median length of 400 to 500 bp using the Covaris M220 instrument. Sheared DNA was purified, and primer extension was performed using a long terminal repeat (LTR) specific biotinylated primer. The extension product was again purified, followed by magnetic capture of the biotinylated DNA for at least 60 minutes and two washing steps with 100 µl H_2_O. The captured DNA was ligated to linker cassettes including a sample barcode. The ligation product was divided into 2 aliquots and amplified in a first exponential PCR using biotinylated vector- and sequencing adaptor-specific primers. Biotinylated PCR-products were magnetically captured, products were pooled, washed and 1/2 of this eluate served as template for amplification in a second exponential PCR step with primers allowing deep sequencing by MiSeq technology (Illumina) after purification. Preparation for deep sequencing was previously described (17, 18). DNA double barcoding was applied to allow parallel sequencing of multiple samples in a single sequencing run while minimizing sample cross-contamination.

IS were recovered using the S-EPTS/LM-PCR protocol (16) and analyzed using the GENE-IS tool suite (19). Briefly, raw sequence data were trimmed according to sequence quality (Phred 30). Only sequences showing complete identity in both sample barcodes, linker cassette barcode and sequencing barcodes, were further analyzed.

Modern sequencing technologies like Illumina MiSeq allow for semi-quantitative estimation of clonal size by the determination (‘counting’) of the number of retrieved sequences (retrieval frequency) for individual vector-genome junctions (ISs). The relative sequence count of all detected ISs was calculated in relation to all sequences which could be mapped to a definite position in the genome. The ten most prominent ISs (Rank 1: highest sequence count; Rank 2: 2^nd^ highest sequence count; Rank 10: 10^th^ highest sequence count) were analyzed for each sample.

### Histopathology

Thymus samples were formalin-fixed and paraffin-embedded. Sections (1.5-μm) were stained with hematoxylin and eosin, cytokeratin 5 or CD3 antibodies. Digital images were acquired with an Olympus DP70 camera mounted on an Olympus BX60 microscope by using CellF Imaging software (Soft Imaging System GmbH, Munster, Germany). Morphometric analysis of the medullary-to-cortical ratio was evaluated by using Image-Pro software.

### Statistical analysis

Statistical analyses were performed with GraphPad Prism software (GraphPad Software) by using Spearman correlation or nonparametric 1-way ANOVA Kruskal-Wallis test. Dunn’s multiple comparisons test was performed after ANOVA, comparing GT groups to the correspondent mutant controls and BMT WT mice, unless otherwise stated. Statistical differences between autoantibody profiles were assessed by global test (20) using the GlobalAncova R package. For all tests, a *p* value of less than 0.05 was considered significant. Further details on significance levels are provided in figure legends.

## Results

### Gene therapy preserves HSPC clonogenic potential and does not impact mouse survival

Lentiviral vector (LV) gene therapy has been shown to obtain effective *RAG1* expression and immune reconstitution in *Rag1^-/-^* mice using a MND-c.o.RAG1 vector (10). We extensively tested the same LV in the setting of 2 mouse models carrying hypomorphic *Rag1* mutations and recapitulating the CID phenotype, the *Rag1^F971L/F971L^* and the *Rag1^R972Q/R972Q^* mice (15). To this purpose, we isolated Lineage-negative (Lin-) cells from the bone marrow (BM) of donor animals and transduced them with the MND-c.o.RAG1 LV. Transduced Lin- cells showed clonogenic potential similar to untreated Lin- cells (**Supplementary Figure S1A**) indicating that LV transduction does not affect stemness potential. Vector copy number/genome (VCN) evaluated in transduced bulk population ranged from 1.2 to 4.0 (**Supplementary Figure S1B**), while VCN in single colony forming units had a median of 2.1 in *Rag1^F971L/F971L^* and 1.4 in *Rag1^R972Q/R972Q^* gene therapy (GT) cells (**Supplementary Figure S1C**), with a mean of transduced colonies of 87.1% and 76.8%, respectively (**Supplementary Figure S1D**).

For the *in vivo* experiments, GT-treated animals received transduced cells after lethal TBI. A total of 23 *Rag1^F971L/F971L^* and 29 *Rag1^R972Q/R972Q^* mice received the corresponding GT cells, while controls were transplanted with wild-type (WT) Lin- cells (BMT WT) or untransduced mutant Lin- cells (BMT UT). Four experiments were terminated 6 months after transplant and two experiments 12 months after transplant. The survival curve of 6-month experiments showed 84% survival in *Rag1^F971L/F971L^* GT and 90% in *Rag1^R972Q/R972Q^* GT mice. Early loss (2 weeks after transplant) of BMT UT *Rag1^R972Q/R972Q^* mice was observed, probably due to lack of engraftment of transplanted cells (**Supplementary Figure S2A**). In experiments terminated at 12 months, we experienced long-term loss of BMT UT animals in both mutant strains, probably due to disease progression exacerbated by the irradiation procedure. Survival of 100% *Rag1^F971L/F971L^* GT and 90% *Rag1^R972Q/R972Q^* GT mice was observed, in line with the experiments terminated 6 months post-treatment (**Supplementary Figure S2B**).

### Gene therapy ensures partial immune reconstitution

**Figure 1:**
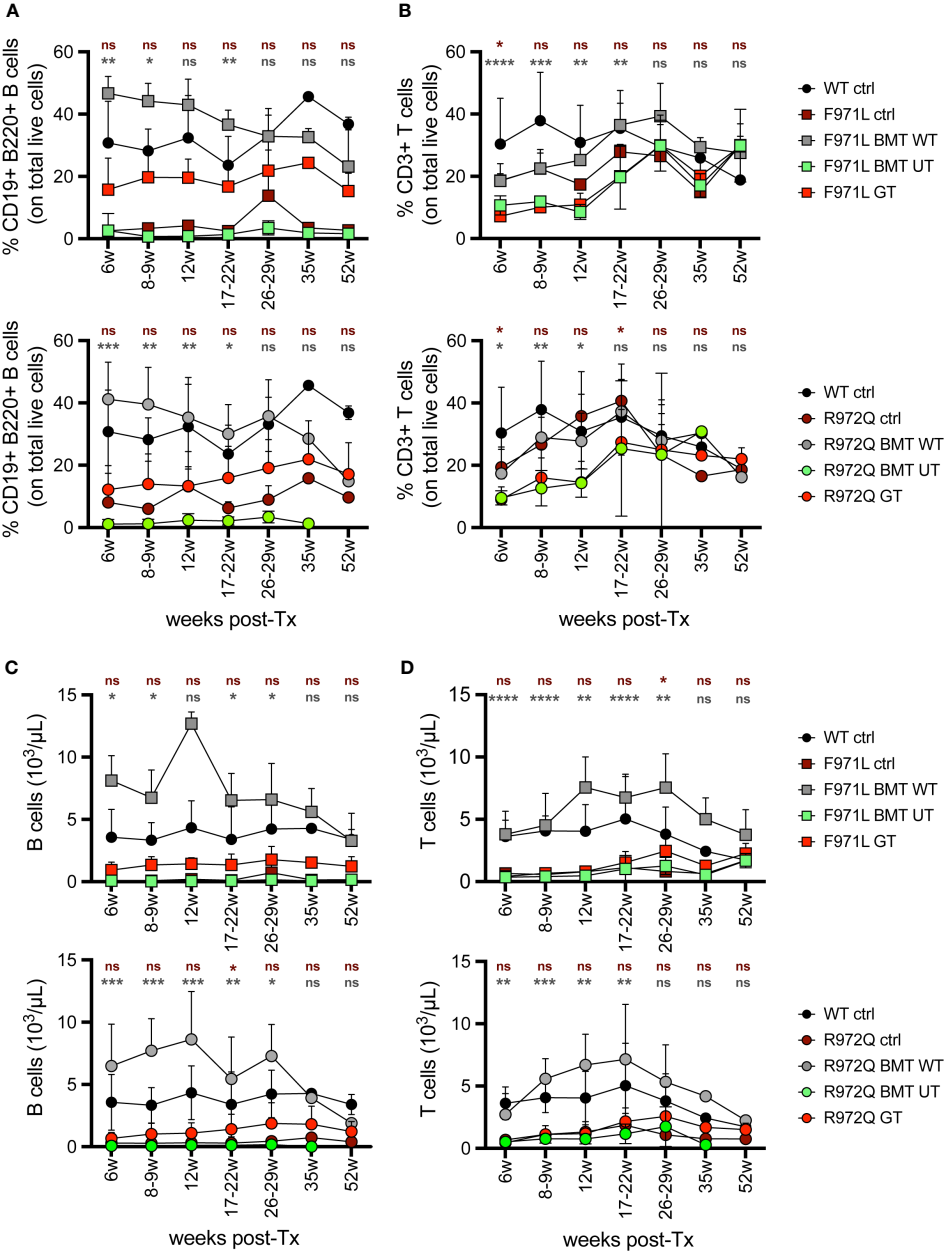
Lymphocyte frequency and counts in peripheral blood. **(A, B).** Relative proportion of B **(A)** and T **(B)** cells were analyzed over time in the peripheral blood of *Rag1^F971L/F971L^* (square symbols, upper panel) and *Rag1^R972Q/R972Q^* (round symbols, lower panels). **(C, D)**. Absolute counts of B **(C)** and T **(D)** cells in the peripheral blood of *Rag1^F971L/F971L^* (square symbols) and *Rag1^R972Q/R972Q^* (round symbols). WT ctrl, wild-type control (n=2-12); F971L ctrl, untreated *Rag1^F971L/F971L^* (n=1-8); R972Q ctrl, untreated *Rag1^R972Q/R972Q^* (n=1-8); F971L BMT WT, *Rag1^F971L/F971L^* transplanted with WT cells (n=2-6); R972Q BMT WT, *Rag1^R972Q/R972Q^* transplanted with WT cells (n=1-9); F971L BMT UT, *Rag1^F971L/F971L^* transplanted with untransduced cells (n=2-8); R972Q BMT UT, *Rag1^R972Q/R972Q^* transplanted with untransduced cells (n=1-8); F971L GT, *Rag1^F971L/F971L^* transplanted with gene therapy cells (n=4-23); R972Q GT, *Rag1^R972Q/R972Q^* transplanted with gene therapy cells (n=5-29). Graphs show mean ± standard deviation (SD). n=6 independent experiments. Statistical analysis: non parametric one-way ANOVA, *p<0.05, **p<0.01, ***p<0.001, ****p<0.0001, ns not significant p>0.05; in red are shown differences between GT and mutated *Rag1* controls, in grey between GT and BMT WT.

In the peripheral blood (PB), analysis of immune reconstitution over time showed a partial increase in the percentage of B cells in *Rag1^F971L/F971L^* GT mice compared to *Rag1^F971L/F971L^* untreated control and BMT UT mice. However, the relative frequency after GT remains significantly lower than that achieved in *Rag1^F971L/F971L^* BMT WT mice, up to 22 weeks post treatment (**Figure 1A**). On the other hand, the low frequency of T cells found in untreated control mice did not improve after GT and remained significantly lower than mice transplanted with WT cells (**Figure 1B**). At later time points, no statistically significant differences were found in the frequency of B and T cells, probably due to the myeloid skewing of aging mice and the lower number of mice tested starting from 35 weeks post-GT. Similar results were found in the relative frequency of B and T cells of *Rag1^R972Q/R972Q^* GT mice (**Figures 1A, B**). Concomitantly, GT mice from both strains had significantly higher proportion of myeloid cells at earlier time points (**Supplementary Figure S3A**). However, B, T and total white blood cell absolute counts remained comparable to untreated *Rag1* mutated controls and significantly lower than WT controls and BMT WT mice (**Figures 1C, D** and **Supplementary Figure S3E**). No major changes were observed in myeloid and natural killer (NK) absolute counts (**Supplementary Figures S3B-D**).

**Figure 2:**
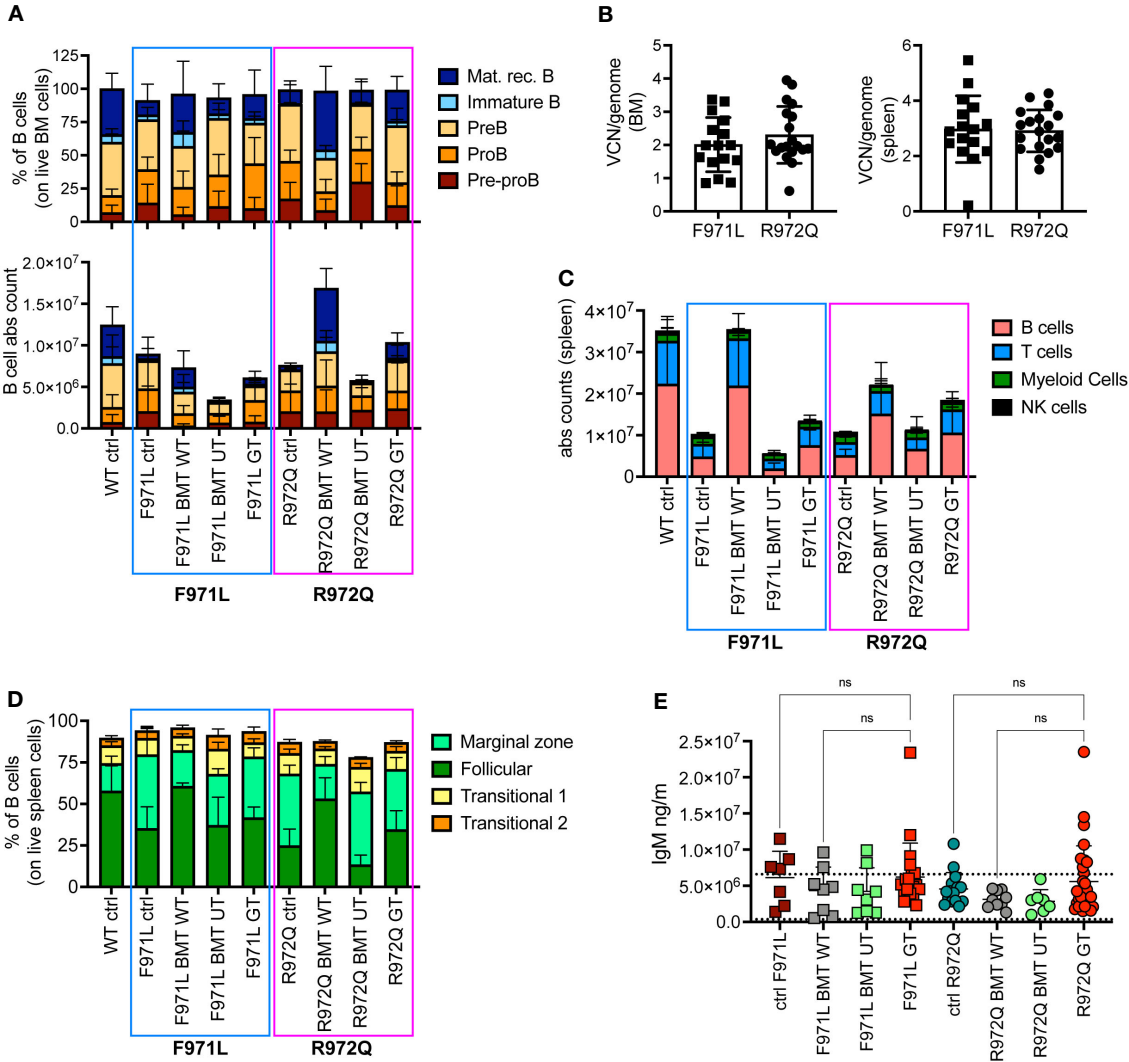
B cell reconstitution at termination 6 months post-transplant. **(A)** B cell subsets in bone marrow (BM) of treated and control (ctrl) mice, shown as relative frequency (upper panel) and absolute counts (lower panel). **(B)** Vector copy number (VCN)/genome in single cell suspensions from bone marrow (left) and spleen (right) of hypomorphic gene therapy (GT) mice. **(C)** Absolute counts of B, T, myeloid and natural killer (NK) cells in the spleen of control and treated mice. **(D)** Frequency of follicular, marginal zone and transitional B cells of the spleen. **(E)** Immunoglobulin M (IgM) levels in plasma. Dotted lines indicate the range of IgM levels in untreated age-matched WT mice. WT ctrl, wild-type control [n=15 **(A-D)**, n=18 **(E)**; F971L ctrl, untreated *Rag1^F971L/F971L^* (n=7); R972Q ctrl, untreated *Rag1^R972Q/R972Q^* (n=11 **(A-D)**, n=15 **(E)**; F971L BMT WT, *Rag1^F971L/F971L^* transplanted with WT cells (n=4 **(A-D)**, n=8 **(E)**]; R972Q BMT WT, *Rag1^R972Q/R972Q^* transplanted with WT cells [n=6 **(A-D)**, n=9 **(E)**]; F971L BMT UT, *Rag1^F971L/F971L^* transplanted with untransduced cells [n=5 **(A-D)**, n=8 panel **(E)**]; R972Q BMT UT, *Rag1^R972Q/R972Q^* transplanted with untransduced cells (n=5 **(A-D)**, n=7 **(E)**; F971L GT, *Rag1^F971L/F971L^* transplanted with gene therapy cells [n=16 **(A-D)**, n=20 **(E)**]; R972Q GT, *Rag1^R972Q/R972Q^* transplanted with gene therapy cells [n=19 **(A-D)**, n=29 **(E)**. n=4 independent experiments. Graphs show mean ± SD. Statistical analysis **(E)**: non parametric one-way ANOVA, ns, not significant p>0.05.

At euthanasia 6 months after treatment, we assessed the immune reconstitution and VCN in hematopoietic organs. B cell lymphopoiesis in bone marrow (BM) revealed the appearance of immature B cells and mature recirculating B cells in both hypomorphic mutants after GT, although absolute counts remained lower than BMT WT mice (**Figure 2A** and **Supplementary Figure S4**). In the *Rag1^R972Q/R972Q^* GT mice, statistically significant higher counts of immature and mature recirculating B cells than untreated control mice were observed, whereas no significant differences were found in *Rag1^F971L/F971L^* GT when compared to both untreated and BMT WT groups (**Supplementary Figures S4F-J**). Mean VCN in BM was 2.0 and 2.8 in *Rag1^F971L/F971L^* and *Rag1^R972Q/R972Q^* GT mice, respectively (**Figure 2B**). In the spleen, some improvements of B and T cell counts were observed in GT mice, but no statistically significant differences were found between GT groups and BMT WT or untreated control mice (**Figure 2C** and **Supplementary Figures S5A-D**). GT induced a modest increase in the proportion of follicular B cells, especially in the *Rag1^R972Q/R972Q^* model, and a slight decrease of marginal zone B cells, which were elevated in untreated or BMT UT mutant mice (**Figure 2D**). No significant differences were found, except for *Rag1^R972Q/R972Q^* GT mice that had increased proportion of marginal zone B cells when compared to BMT WT counterpart (**Supplementary Figures S5E-H**). In addition, we observed a trend to increase the white pulp area after GT, in line with that of BMT WT controls (**Supplementary Figure S5I**). To assess the functionality of B cells we quantified immunoglobulin (Ig) levels in the plasma. IgM serum levels were increased in a fraction of mutant mouse, but no statistically significant differences were detected (**Figure 2E**). Similarly, no major differences were observed in IgA and IgG levels in the *Rag1^F971L/F971L^* strain, while in the *Rag1^R972Q/R972Q^* GT mice a statistically significant increase of IgA after GT was observed (**Supplementary Figures S5J, K**).

**Figure 3:**
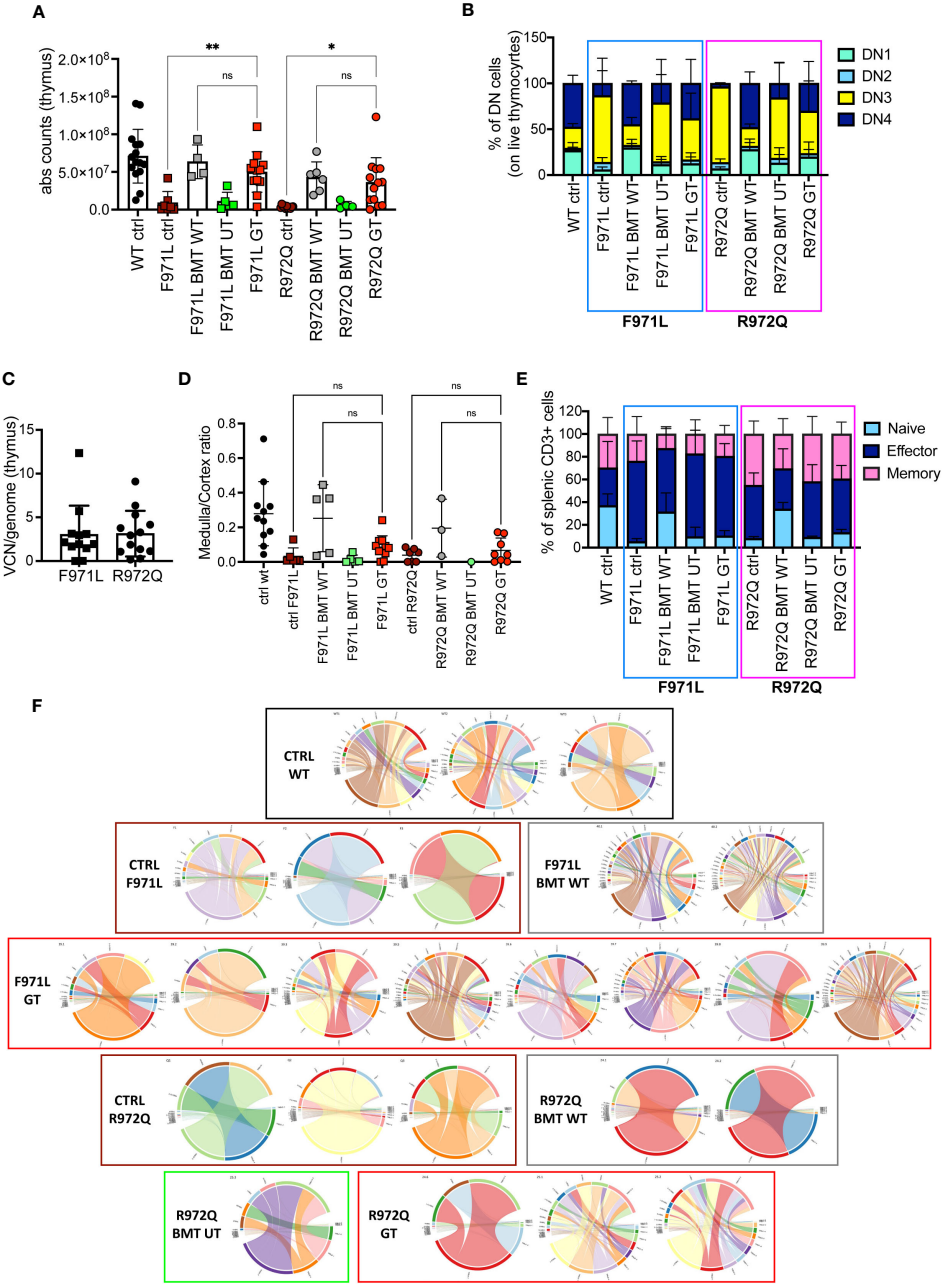
T cell reconstitution at termination 6 months post-transplant. **(A)** Total counts of thymocytes. **(B)** Frequency of double negative (DN) stages 1-4 of thymocytes. **(C)** Vector copy number (VCN)/genome in single cell suspensions from thymus of hypomorphic gene therapy (GT) mice. **(D)** Ratio between medulla and cortex areas, evaluated by hematoxylin and eosin staining on thymic tissues. **(E)** Frequency of naïve, effector and memory T cells of the spleen. **(F)** V- and J-gene recombination of T cell receptors (TCR) in splenocytes. Each bow between V- and J-gene represents a clonotype. The broader a bow, the higher relative frequency was detected for the respective clonotype. WT ctrl, wild-type control (n=11-15); F971L ctrl, untreated *Rag1^F971L/F971L^* (n=6-7); R972Q ctrl, untreated *Rag1^R972Q/R972Q^* (n=7-11); F971L BMT WT, *Rag1^F971L/F971L^* transplanted with WT cells (n=5); R972Q BMT WT, *Rag1^R972Q/R972Q^* transplanted with WT cells (n=3-6); F971L BMT UT, *Rag1^F971L/F971L^* transplanted with untransduced cells (n=5); R972Q BMT UT, *Rag1^R972Q/R972Q^* transplanted with untransduced cells (n=1-5); F971L GT, *Rag1^F971L/F971L^* transplanted with gene therapy cells (n=11-13); R972Q GT, *Rag1^R972Q/R972Q^* transplanted with gene therapy cells (n=8-19 panels). n=4 (panels **(A–C, E)**, n=3 **(D)** or n=1 **(F)** independent experiments. Graphs show mean ± SD. Statistical analysis: non parametric one-way ANOVA **(A, D)**: *p<0.05, **p<0.01, ns not significant p>0.05.

In the thymus, normalization of total thymocyte counts was observed after GT (**Figure 3A**). GT-treated mice displayed partial correction of the block at double negative (DN) 3 stage, especially in the *Rag1^R972Q/R972Q^* GT mice (**Figure 3B** and **Supplementary Figures S6A-D**). VCN was variable and ranged from 0.01 to 12.3, although comparable in the 2 mutant GT groups (**Figure 3C**). Histological and immunohistochemistry evaluation confirmed partial thymic immune reconstitution, with modest improvement in the cortical/medullary ratio of some GT mice (**Figure 3D** and **Supplementary Figures S7A, B**). However, statistically significant correlation with the VCN of thymocytes was found only in the *Rag1^R972Q/R972Q^* strain (**Supplementary Figure S7C**). In the periphery, phenotypic analysis of splenic T cells demonstrated no significant changes in the proportion of naïve, effector and memory T cells in GT treated mice as compared to BMT UT mice, as shown by persistence of an elevated fraction of effector T cells and a reduced proportion of naïve T lymphocytes (**Figure 3E** and **Supplementary Figures S6E-G**). We assessed the T cell receptor (TCR) clonality from the splenocytes of treated mice, 6 months post-treatment. In *Rag1^F971L/F971L^* strain, the lowest number of clonotypes and diversity values could be seen in the untreated control mice, whereas mice from the BMT WT group showed the highest V- and J-gene recombination. In the GT group, variable recombination was observed ranging from low to high TCR clonality. In *Rag1^R972Q/R972Q^* mice, similar results were obtained, although BMT WT mice showed an oligoclonal repertoire. One GT mouse showed low V/J recombination, while 2 out of 3 GT mice had high diversity values (**Figure 3F**).

**Figure 4:**
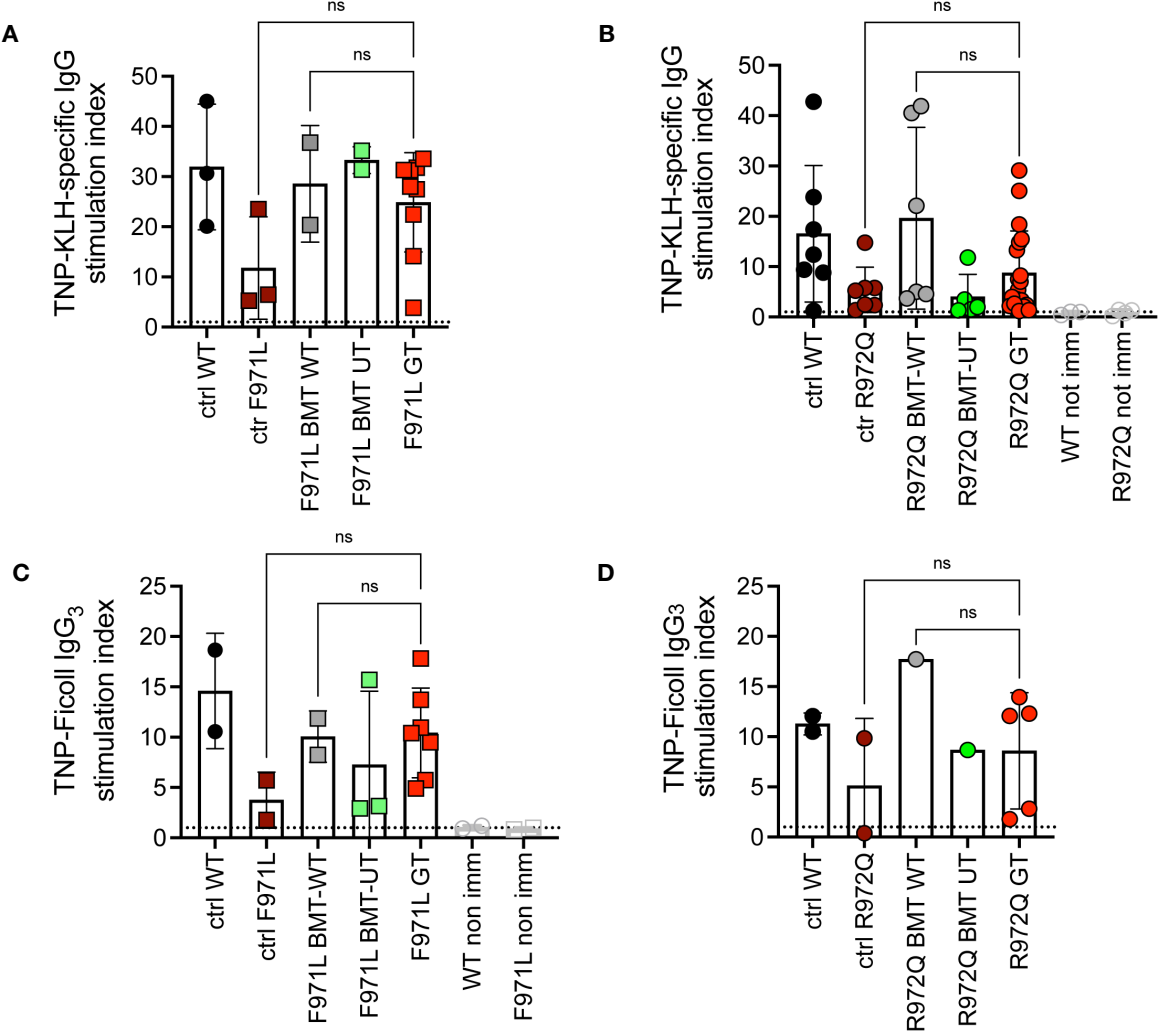
*In vivo* response to antigens. **(A, B).** IgG response specific to trinitrophenyl-keyhole limpet hemocyanin (TNP-KLH) antigen was evaluated by ELISA and shown as stimulation index comparing values at day 28 (7 days after the boosting dose) and day 0 in *Rag1^F971L/F971L^* **(A)** and *Rag1^R972Q/R972Q^* **(B)** mice. **(C, D).** IgG_3_ response specific for TNP-Ficoll antigen, shown as stimulation index comparing values at day 14 and day 0. WT ctrl, wild-type control (n=2-7); F971L ctrl, untreated *Rag1^F971L/F971L^* (n=2-3); R972Q ctrl, untreated *Rag1^R972Q/R972Q^* (n=2-7); F971L BMT WT, *Rag1^F971L/F971L^* transplanted with WT cells (n=2); R972Q BMT WT, *Rag1^R972Q/R972Q^* transplanted with WT cells (n=1-6); F971L BMT UT, *Rag1^F971L/F971L^* transplanted with untransduced cells (n=2-3); R972Q BMT UT, *Rag1^R972Q/R972Q^* transplanted with untransduced cells (n=1-5); F971L GT, *Rag1^F971L/F971L^* transplanted with gene therapy cells (n=7-9); R972Q GT, *Rag1^R972Q/R972Q^* transplanted with gene therapy cells (n=5-19). Graphs show mean ± SD, dotted line at stimulation index = 1. Statistical analysis: non parametric one-way ANOVA: ns, not significant p>0.05. n=1 **(A, C, D)** or n=3 independent experiments **(B)**.

To understand if this incomplete immune reconstitution, compared to mice treated with WT stem cells, would suffice to induce adaptive immune responses, we assessed antibody responses upon challenge with T-dependent (TNP-KLH) and T-independent (TNP-Ficoll) antigens. The TNP-KLH-specific response was evaluated by specific IgG production after the second boosting dose. In *Rag1^F971L/F971L^* mice, we did not detect differences in TNP-KLH-specific IgG serum levels among the various experimental groups (**Figure 4A**). In *Rag1^R972Q/R972Q^* treated mice, the improvement in specific IgG response was comparable to WT or BMT WT animals, although no significant differences were found, also due to the variability of the assay (**Figure 4B**). In parallel, another cohort of animals were immunized with the T-independent antigen. Levels of TNP-specific IgG_3_ were similar in GT mice, and WT controls (**Figures 4C, D**).

**Figure 5:**
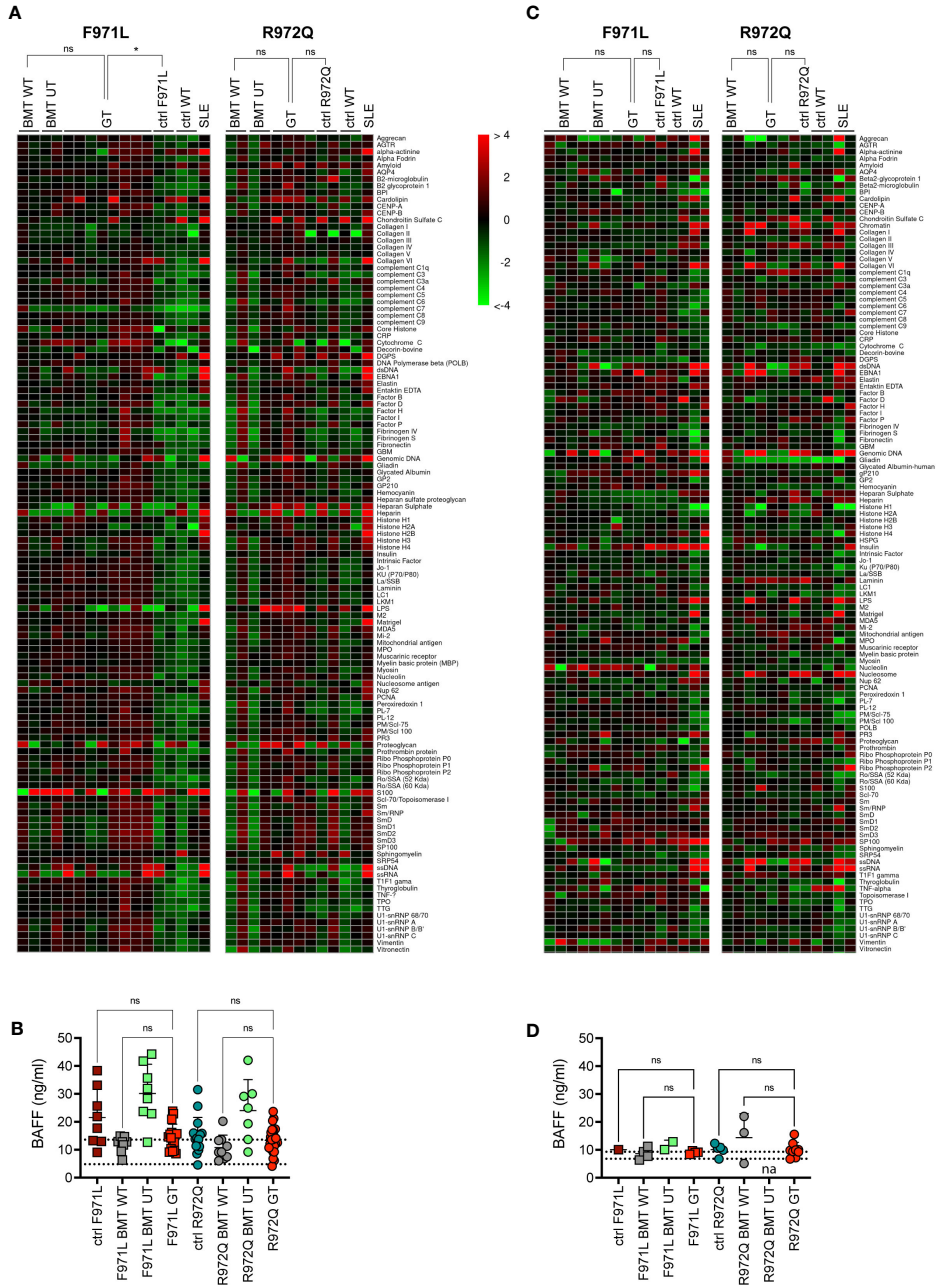
Autoimmune manifestations. **(A)** Heatmap of serum IgG autoantibodies in serum at 6 months post-transplant, normalized to BMT WT reactivity. The color scale corresponds to the fold-change in expression on the log2 scale, using BMT WT as a reference. **(B)** B-cell activating factor (BAFF) levels in serum 6 months post-transplant. Dotted lines indicate the range of BAFF levels assessed in untreated age-matched WT mice. **(C)** Heatmap of serum IgG autoantibodies in serum at 12 months post-transplant, normalized to BMT WT reactivity. **(D)** B-cell activating factor (BAFF) levels in serum 12 months post-transplant. Dotted lines indicate the range of BAFF levels assessed in untreated age-matched WT mice. SLE, systemic lupus erythematosus. WT ctrl, wild-type control (n=4-20); F971L ctrl, untreated *Rag1^F971L/F971L^* (n=1-8); R972Q ctrl, untreated *Rag1^R972Q/R972Q^* (n=2-15); F971L BMT WT, *Rag1^F971L/F971L^* transplanted with WT cells (n=2-8); R972Q BMT WT, *Rag1^R972Q/R972Q^* transplanted with WT cells (n=2-9); F971L BMT UT, *Rag1^F971L/F971L^* transplanted with untransduced cells (n=2-8); R972Q BMT UT, *Rag1^R972Q/R972Q^* transplanted with untransduced cells (n=2-7); F971L GT, *Rag1^F971L/F971L^* transplanted with gene therapy cells (n=4-17); R972Q GT, *Rag1^R972Q/R972Q^* transplanted with gene therapy cells (n=5-29). n=1 **(A, C, D).** or n=4 **(B).** independent experiments. Graphs show mean ± SD **(B, D).**. Statistical analysis: Global Ancova test **(A, C).** or non parametric one-way ANOVA **(B, D).**. ns, not significant p>0.05; *p<0.05.

Since incomplete or ectopic *RAG1* expression could possibly lead to autoimmune manifestations typical of the CID-G/AI phenotype, we assessed the production of autoantibodies in the serum. Autoantibodies were tested at 6 months post-transplant, by screening a panel of 123 autoantigens using a high-throughput autoantigen microarray platform. No increase of IgG autoantibodies was observed after GT, when values were normalized to the correspondent BMT WT controls. Statistical analysis, despite the low number of analyzed mice, showed a statistical difference between *Rag1^F971L/F971L^* controls and GT animals, while no differences were found between GT and BMT WT groups of both strains (**Figure 5A**). We also measured the levels of the B cell-activating factor (BAFF), a survival and maturation factor for B cells, which is usually upregulated in lymphopenic conditions and autoimmune diseases (21). We observed high BAFF levels in untreated mutant mice, as well as in BMT UT animals, as expected. We detected lower BAFF levels in most *Rag1^F971L/F971L^* and *Rag1^R972Q/R972Q^* GT mice (**Figure 5B**). To exclude late onset of autoimmunity we also measured autoantibody production and BAFF levels 12 months after transplant. We did not observe the increase in autoantibody production in neither *Rag1^F971L/F971L^* nor *Rag1^R972Q/R972Q^* GT mice (**Figure 5C**), in line with those obtained at 6 months post GT. No significant differences between groups were found in BAFF levels (**Figure 5D**), probably due to the general reduction of B cells and the concomitant myeloid skewing in the BM that is expected in aged mice (**Supplementary Figure S8**). It is worth mentioning that mice living in specific pathogen-free condition are not regularly exposed to new antigens and may not efficiently predict the onset rate of autoimmunity in humans.

At 12 months post GT, we confirmed a partial overcoming of the DN3 block in thymocytes of GT animals (**Supplementary Figure S9**). In the spleen, comparable B and T cell counts were observed in GT animals when compared to BMT WT mice, but also to untreated *Rag1^F971L/F971L^* or *Rag1^R972Q/R972Q^* controls, suggesting a general flattening of differences due to the age (**Supplementary Figures S10A-F**). In line with the previous results, TCR repertoire showed lower clonality than at 6 months not only in GT mice, but also in transplanted or untreated controls (**Supplementary Figure S10G**).

### T cells show slower kinetics of reconstitution

To distinguish donor (GT) and recipient cells, we generated hypomorphic *Rag1^R972Q/R972Q^* CD45.1 mice. We transduced donor CD45.1 cells with the MND-c.o.RAG1 vector and transplanted them into CD45.2 mutant *Rag1* recipients, similarly to previous experiments. The mismatched transplants allowed to study the immune reconstitution of GT cells in the setting of hypomorphic RAG1 expression that may favor the persistence of residual T cells after irradiation.

**Figure 6:**
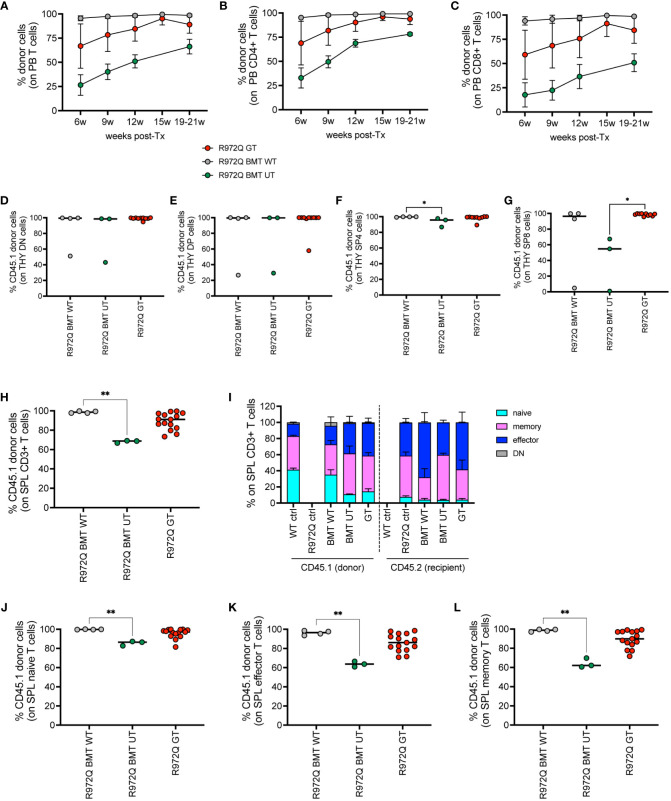
Donor chimerism in CD45-mismatched transplants, terminated 6 months post-transplant. **(A-C).** Chimerism of donor cells (CD45.1) over time in the total T cells **(A)**, CD4+ T cells **(B)** and CD8+ T cells **(C)** of peripheral blood. (**D-G).** Chimerism of CD45.1+ donor cells in the double negative (DN, **(D)**, double positive (DP, **(E)**, single positive 4 (SP4, **(F)** and single positive 8 (SP8, **(G)** subsets of the thymus. **(H)** Chimerism of donor cells in the T cells of the spleen. **(I)** Distribution of naïve, memory, effector and double negative (DN, CD44- CD62L-) T cells of the spleen in donor (CD45.1) and recipient (CD45.2) cells. **(J-L).** Chimerism of CD45.1+ donor cells in the naive **(J)**, effector **(K)**, memory **(L)** splenic T cell subsets. WT ctrl, wild-type control (n=8); R972Q ctrl, untreated *Rag1^R972Q/R972Q^* (n=8); R972Q BMT WT, *Rag1^R972Q/R972Q^* transplanted with WT cells (n=4); R972Q BMT UT, *Rag1^R972Q/R972Q^* transplanted with untransduced cells (n=3); R972Q GT, *Rag1^R972Q/R972Q^* transplanted with gene therapy cells (n=10-15). n=2 independent experiments. Graphs show mean ± SD **(A, C, I)** or median **(D-H, J-L)**. Statistical analysis **(D-H, J-L)**: non parametric one-way ANOVA, *p<0.05, **p<0.01; all other comparisons (not shown) are not significant p>0.05.

We analyzed the engraftment of donor GT cells in PB over time, in comparison to BMT WT and BMT UT mice. We observed full donor chimerism of B, myeloid and NK cells over time in all treated groups (**Supplementary Figures S11A-C**). However, mixed chimerism of T cells was observed, especially in GT and BMT UT treated mice (**Figure 6A**). At 6 weeks, CD4+ T cells had a mean donor engraftment of 69% and 33% in GT and BMT UT, respectively, while BMT WT mice showed 95% chimerism. At 20 weeks, engraftment increased up to 94% and 78% in GT and BMT UT respectively, reaching full chimerism (99%) in BMT WT mice (**Figure 6B**). A similar trend was observed in CD8+ T cells with a mean donor engraftment of 59% and 18% at 6 weeks in GT and BMT UT, respectively, while BMT WT mice showed 94% chimerism. Engraftment increased up to 84% and 50% in GT and BMT UT respectively, reaching full chimerism (99%) in BMT WT mice at 20 weeks (**Figure 6C**).

At 6 months post-transplant, we verified the engraftment of transplanted cells in the hematopoietic organs. In thymus, we found full chimerism in most of GT mice in DN, as well as in double positive (DP) cells (**Figures 6D-E**). In single positive CD4+ and CD8+ cells, lower chimerism was observed only in BMT UT group, while GT animals showed full donor chimerism (**Figures 6F-G**). In the spleen, we confirmed significantly lower chimerism of T cells in BMT UT mice and variable frequency of CD45.1 in GT mice (**Figures 6H**). T cell phenotype confirmed that the majority of naïve T cells were derived from the transplanted cells in all treated groups (**Figures 6I-L** and **Supplementary Figures S11D-I**). Regarding the B cell progenitors in the BM, we found statistically significant lower chimerism in BMT UT in the most primitive pre-proB cells, while GT mice showed variable proportions of donor cells (**Supplementary Figure S11J**). No differences in chimerism were observed in pro, pre, immature or mature recirculating B cells (**Supplementary Figures S11K-N**). Full chimerism of the hematopoietic stem and progenitor cells Lin- Sca1+ cKit+ (LSK) was retrieved in all transplanted animals (**Supplementary Figure S11O**). In the spleen, B, myeloid and NK cells had full donor chimerism (**Supplementary Figures S11P-R**).

In order to dissect the kinetic of T cell reconstitution, we set up a short-term mismatched experiment in both mouse strains, terminated 3 weeks post-transplant. Peripheral blood showed partial T cell chimerism in BMT WT mice and even lower in BMT UT and GT mice from both mutant strains (**Supplementary Figures S12A-C**), although we do not expect T cell reconstitution at this early time point. We found full donor chimerism in all transplanted groups in the DN CD4- CD8- cells of the thymus (**Supplementary Figure S12D**), suggesting that the reduced T cell chimerism in PB is not due to incomplete depletion and/or competition of residual host T cell progenitors in the thymus. On the other hand, partial chimerism was seen in DP and single positive thymocytes, especially in the BMT UT group (**Supplementary Figures S12E-G**). Variability in chimerism is not correlated with VCN (**Supplementary Figure S12H**). These results points to slower T cell differentiation and thymic output when RAG1 expression is reduced or unregulated. Moreover, this suggests an important difference from the *Rag1^-/-^* setting, that benefits from strong selective advantage of GT cells. Chimerism in the spleen was lower in BMT UT and GT mice than in BMT WT, similarly to PB (**Supplementary Figure S12I**). Rag1^F971L/F971L^ GT mice showed a higher VCN in the spleen than Rag1^R972Q/R972Q^ GT mice, despite the lower proportion of donor T cells (**Supplementary Figure S12J**). Notably, we also detected high proportion of CD4- CD8- T cells in the spleen of transplanted mice (**Supplementary Figures S12K-N**), suggesting the presence of immature T cells at this early time-point.

### Abnormal thymic growth in GT mice

**Figure 7:**
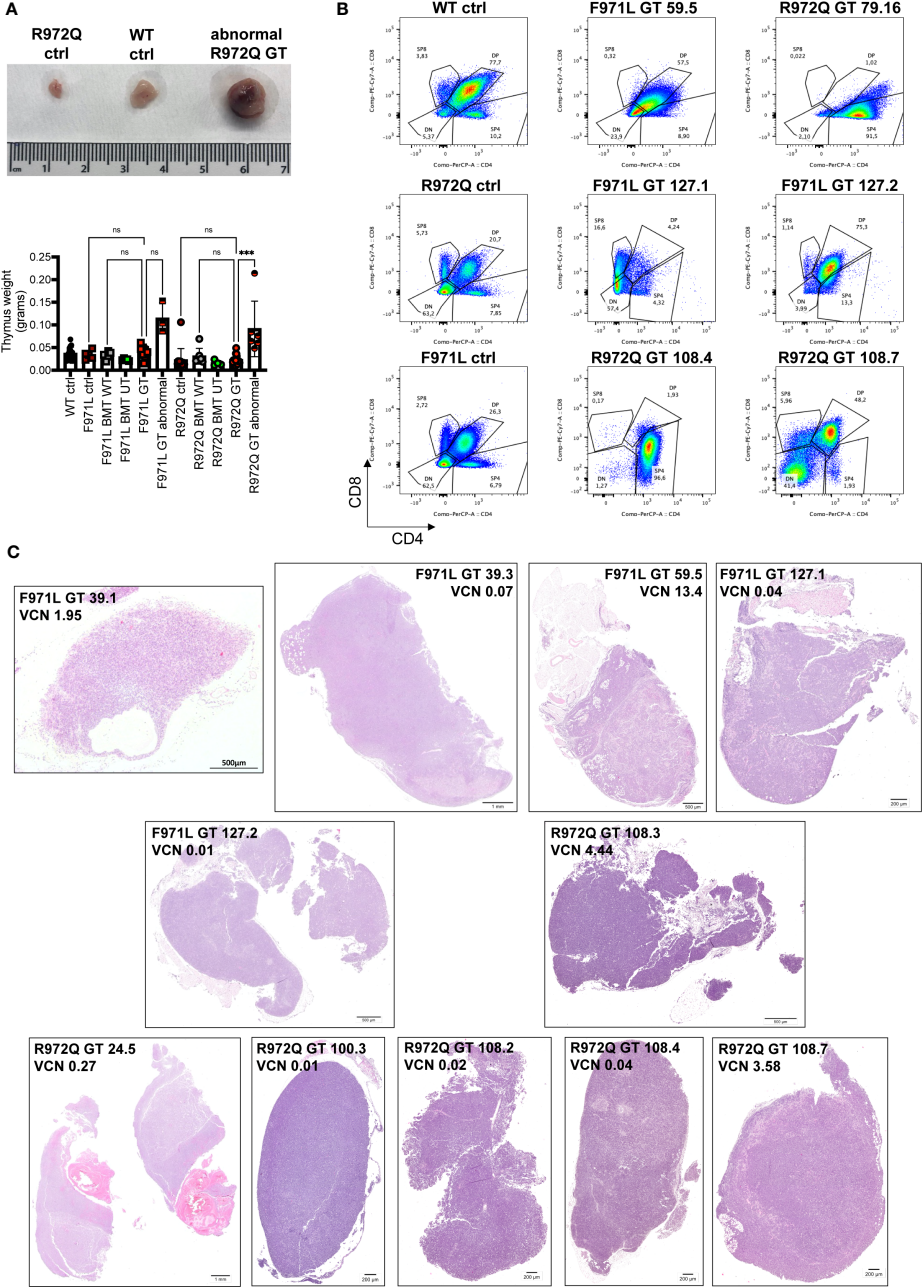
Abnormalities found at euthanasia. **(A)** Upper panel: Representative macroscopic image of enlarged thymus found in a R972Q GT mouse, in comparison with untreated *Rag1^R972Q/R972Q^* and WT controls. Lower panel: graph shows the weight of normal and abnormally big thymi. **(B)** Representative plots of thymocyte subsets after CD4 and CD8 staining, gated on live CD45+ cells, of untreated controls and abnormal GT thymi, terminated at 6 or 12 months post-GT. DN = double negative CD4- CD8- cells; DP = double positive CD4+ CD8+ cells; SP4 = single positive CD4; SP8 = single positive CD8. **(C)** Representative pictures of hematoxilyn and eosin staining of thymic tissue sections of abnormally big GT thymi. Mouse ID, strain and VCN are indicated. WT ctrl, wild-type control (n=17); F971L ctrl, untreated *Rag1^F971L/F971L^* (n=5); R972Q ctrl, untreated *Rag1^R972Q/R972Q^* (n=13); F971L BMT WT, *Rag1^F971L/F971L^* transplanted with WT cells (n=5); R972Q BMT WT, *Rag1^R972Q/R972Q^* transplanted with WT cells (n=7); F971L BMT UT, *Rag1^F971L/F971L^* transplanted with untransduced cells (n=5); R972Q BMT UT, *Rag1^R972Q/R972Q^* transplanted with untransduced cells (n=4); F971L GT, *Rag1^F971L/F971L^* transplanted with gene therapy cells and normal thymus (n=8); F971L GT abnormal, *Rag1^F971L/F971L^* transplanted with gene therapy cells and abnormal thymus (n=3); R972Q GT, *Rag1^R972Q/R972Q^* transplanted with gene therapy cells and normal thymus (n=10); R972Q GT abnormal, *Rag1^R972Q/R972Q^* transplanted with gene therapy cells and abnormal thymus (n=6). n=6 independent experiments. Graph **(A)** shows mean ± SD. Statistical analysis **(A)**: non parametric one-way ANOVA: ns, not significant p>0.05; ***p<0.001.

At termination, 6- or 12-months post-transplant, a total of 4 out of 20 (20%) *Rag1^F971L/F971L^* and 7 out of 28 (25%) *Rag1^R972Q/R972Q^* GT mice showed an abnormally enlarged thymus (**Figure 7A**). In addition, 1 *Rag1^F971L/F971L^* GT mouse showed a cystic formation in a thymus of normal size. To characterize these abnormalities, we performed histological, cellular (flow cytometry) and molecular (vector copy number and integration site analysis) assays (**Table 1**). Flow cytometry analyses showed different expanded populations (**Figure 7B**), with a disorganized thymic tissue being a recurrent histological finding in most of them (**Figure 7C**). VCN detected in these tissues (**Table 1**), although variable, was in line with those observed in normal thymi (**Figure 3C** and **Supplementary Figure S9F**), suggesting that the LV is not involved in transformation. This observation is also supported by the very low VCN retrieved in 3 samples (**Table 1**, mouse ID: 39.3, 127.1 and 127.2 mice).

**Table 1:** List of abnormalities found at termination.

experiment (termination point)	mouse no.	strain	thymus	VCN	Histological analysis	Flow cytometry	Integration site analysis
GT8 (6M)	39.1	F971L	small with a cystic formation	1.95	Immature thymus with cystic formation. No medullary area. Widespread CD3+ cells	not done	Top10 integrations 98.17%. The two most dominant IS: 53.91% Sik3 and 24.56% Brd4
GT8 (6M)	39.3	F971L	abnormal, big	0.07	Normal tissue in a small portion, most tissue similar to thymoma, few CD3+ cells	not done	Top10 integrations 75.33%. Two most dominant IS: 20.935% Arpp21 and 15.15% Maml1
GT8 (6M)	24.5	R972Q	abnormal, big	0.27	Cystic formation and tissue similar to thymoma. No medullary area	not done	Top10 integrations 95.69%. Three most dominant IS: 33.14% Ofd1, 25.03% Mir195b, 23.22% Samm50
GT9 (12M)	59.5	F971L	abnormal, big	13.4	Tissue similar to thymoma with adipose tissue. No medullary area. Widespread CD3+ cells	71.2% DP cells	Top10 integrations 83.93%. Oligoclonal IS composition with top IS 11.07% Atad2
GT10 (12M)	79.16	R972Q	abnormal, big	0.13	not available	98.1% CD4 SP	Top10 integrations 53.91%. Polyclonal IS composition with top IS 12.54% Chd1
GT11 (6M)	127.1	F971L	abnormal, big	0.04	Tissue similar to thymoma. No medullary area. Widespread CD3+ cells	79.5% DN cells (50.0% DN3 and 47.6% DN4)	Top10 integrations 91.89%. Oligoclonal IS composition with top IS 19.80% Baiap2
GT11 (6M)	127.2	F971L	abnormal, big	0.01	Heterogenous tissue: a portion with disorganized medulla/cortex and low CD3+ cells; another portion similar to thymoma without medulla and widespread CD3+ cells.	90.8% DP cells	Top10 integrations 99.86%. Three most dominant IS: 29.19% Agmo, 21.76% Arhgap35, 20.54% Xrcc5
GT12 (6M)	100.3	R972Q	1 lobe enlarged	0.01	No medullary area. Widespread CD3+ cells.	64% SP8 cells (of which 99% are host- derived)	not done
GT13 (6M)	108.2	R972Q	1 lobe enlarged	0.02	Absence of medullary area in most of the tissue. Widespread CD3+ cells.	95% DP (of which 99.5% host-derived)	not done
GT13 (6M)	108.3	R972Q	1 lobe enlarged	4.44	No medullary area. Widespread CD3+ cells.	90% DP (of which 99.5% donor-derived)	not done
GT13 (6M)	108.4	R972Q	1 lobe enlarged	0.04	Heterogenous tissue: a portion with correctly organized tissue and polarization; another portion similar to thymoma without medulla and widespread CD3+ cells.	97% SP4 (of which 99.6% host-derived)	not done
GT13 (6M)	108.7	R972Q	1 lobe enlarged	3.58	No medullary area. Widespread CD3+ cells.	41% DN and 48% DP (of which 100% donor-derived in both DN and DP)	not done

To better dissect the contribution of LV to tissue transformation, integration site (IS) analysis was performed. Results demonstrated an oligoclonal composition because Top10 IS accounted for most of total IS in both macroscopically abnormal and normal thymic tissues (**Supplementary Figure S13**). Similar profiles, although with a more polyclonal profile (lower cumulative frequency of Top10 IS), was observed in the corresponding spleen cells. IS in proximity to *MECOM* and *LMO2* genes, involved in severe adverse events of previous GT clinical trials (22–29), were found only in 6 samples from 4 mice, with individual retrieval frequencies well below 1% in all cases (**Supplementary Table S1**). Therefore, no direct correlation could be established between abnormal thymic tissue and vector integration, although a formal toxicology study would be required in hypomorphic murine models, where the immune dysregulated context may influence the onset of thymic hyperplasia.

## Discussion

Lentiviral vector gene therapy for the treatment of *RAG*-SCID can benefit from the selective advantage of corrected B and T cells (1), similarly to other diseases (30). In patients affected by hypomorphic RAG1 deficiency, the few residual T and B cells limit the effect of selective advantage (6) and thus a stronger and more regulated transgene expression may be required. In addition, the combined presence of immunodeficiency and immune dysregulation demands caution to the application of innovative treatments, such as gene therapy, that have suboptimal and non-physiological transgene expression. For these reasons, we tested the MND-c.o.RAG1 lentiviral vector, that has been chosen for a phase I/II clinical trial to treat *RAG1*-SCID (10), in two murine models of CID, the *Rag1^F971L/F971L^* and the *Rag1^R972Q/R972Q^* mice, in order to assess the efficacy and safety of the treatment.

Our results show a partial reconstitution of T and B cell frequencies and counts after GT, in line with the data on *Rag1^-/-^* mice treated with the same LV and transduction protocol (10). Notably, the VCN we retrieved in BM and spleen is higher than those reported for *Rag1^-/-^* GT mice, but it does not result in better reconstitution levels (10). A possible explanation for the partial lymphocyte reconstitution is the competition with the defective precursors and the few residual T and B cells, that could limit the engraftment and the selective advantage of transduced cells. In CD45-mismatched transplants, we observed full chimerism in LSK progenitors and B cells in *Rag1^R972Q/R972Q^* GT mice at 6 months post-transplant, while slightly reduced T cell chimerism was observed in blood and spleen but not in the thymus. These results suggest that lymphoid precursors in central organs are efficiently eliminated by irradiation and are not responsible for the partial reconstitution. Concordantly, the short-term experiment (terminated 3 weeks post-treatment) showed full chimerism in LSK cells, B cells and, most importantly, double negative thymocytes, where the competition with *Rag1*-defective immature precursors is higher. Very low peripheral T cell chimerism was observed, probably due to the longer time necessary for T cell engraftment. On the other hand, in long-term experiments residual host T cells were found in the blood of *Rag1^R972Q/R972Q^* GT and BMT UT groups, up to 12 weeks post-GT or to termination, respectively. Because BMT WT show full chimerism since the first analyzed time point (6 weeks), hypomorphic and/or unregulated RAG1 expression could be responsible for the slower reconstitution kinetics.

B cells have historically been more difficult to reconstitute to normal counts after HSCT or GT (31, 32), and some patients required intravenous immunoglobulin administration. In hypomorphic mice, we observed that low B cell counts in periphery are accompanied by normal IgG production before and after GT. Importantly, humoral response to T-dependent or T-independent antigens is comparable to that obtained in BMT WT mice, although not statistically significant results were found. These data indicate that B cell reconstitution, although partial, can be protective from infections.

Another hypothesis for the incomplete lymphocyte reconstitution could be that tight physiological gene regulation is more important than the transgene copy number in the genome. In mice, *RAG* genes have at least 2 waves of expression during both thymocyte and B cell precursor differentiation, to drive the rearrangement of the different chains of TCR and BCR (33, 34). Chromatin accessibility and transcription factors are fundamental to induce the transcriptionally active conformation of the *RAG* locus (35). In addition, the *MND* promoter that ensures sufficient *RAG1* expression in our vector is ubiquitously expressed, so no level of regulation is present, except via the expression of *RAG2*. This lack of gene regulation, that may be partially introduced with transgene-specific promoters, is a well-known limitation of current gene therapy, but actual recombinase activity is well regulated via RAG2 expression. Gene editing approaches might overcome this limitation by precisely introducing the therapeutic transgene into the endogenous locus preserving RAG1 physiological regulation (36, 37). On the other hand, a comprehensive analysis of rescue of RAG1 function is needed, as uncorrected cells might still differentiate into T and B cells in hypomorphic RAG patients. Finally, an assessment of genome integrity upon gene editing is mandatory before moving to the clinical application.

The *MND* is a quite strong promoter and has already been used in previous trials of gamma-retroviral vector GT for adenosine deaminase deficiency (ADA)-SCID (38) and LV GT for adrenoleukodystrophy (ALD) (39, 40). While long-term follow up of ADA-SCID patients showed the safety of the therapy, three cases (out of 67 treated patients) of myelodysplastic syndrome (MDS) due to insertional mutagenesis were recently reported in ALD (40, 41). Integration site analysis demonstrated vector integrations close to *MECOM* and *PRDM16* proto-oncogenes, known for insertional mutagenesis in previous trials (22, 23, 27, 29, 42–44). Our LV has been shown to be reasonably safe when tested by the *in vitro* immortalization (IVIM) assay, the current safety test accepted by regulatory agencies, and also by the newer surrogate assay for genotoxicity assessment (SAGA) assay (45, 46). These *in vitro* assays can predict the mutagenic risk of vectors, although they are not able to consider the complexity of an *in vivo* organism and the disease background. Furthermore, the assessment done in *Rag1^-/-^* mice showed favorable safety data (10) and the treatment of *RAG1*-SCID patients in the clinical trial will provide important human data. Moreover, in the three cases of MDS reported in ALD GT, all malignant clones had 3 to 5 lentiviral integrations including multiple near oncogenes. For this reason, the *RAG1*-SCID clinical trial aimed for low VCN (around 1) but still with sufficient expression to mimic the physiological RAG1 expression levels in the thymus. Indeed, overcoming of a minimal threshold of RAG1 expression (47, 48) is required to achieve efficient T cell development (49).

In this study we found that 25% of GT mice from both strains had enlarged thymic tissue at termination, 6- or 12-months post GT. Vector copy number retrieved in thymocytes was in line with that of thymi of normal size. Integration site analysis showed oligoclonal to polyclonal repertoire in both normal and abnormal samples. In 4 mice, we found IS near *MECOM* or *LMO2* proto-oncogenes at very low retrieval frequency. These data indicate that vector integration should not be responsible for thymic enlargement. Moreover, in mismatched transplant, we demonstrated that 3 out of 5 cases of abnormal thymi were due to the expansion of host cells, supporting the previous observation. The presence of immune dysregulation in *Rag1* hypomorphic mice may have contributed to the thymic enlargement, that was not reported for *Rag1^-/-^* mice. The presence of a rudimental thymus with a strong perturbation of epithelial thymic cells and the presence of both functional and dysfunctional thymocytes may have limited hyperplasia. In the past, onset of T cell leukaemia and enlargement of thymi was shown when few clones sustained T cell reconstitution in X-linked SCID (SCIDX1) mice, that bear mutations in the IL-2 receptor gamma chain (50–52).

Treatment of hypomorphic *RAG* patients is less straightforward than typical SCID. Their autoimmune problems often require immunosuppressive and immunomodulatory drugs, but this only further increases their risk for infections. Patients are often diagnosed at a later age when organ damage has already developed and HSCT becomes more challenging overall. There is an increased risk of developing graft-versus-host disease (GvHD) and the partial development of T and B cells requires the use of myeloablative conditioning regimen to prevent graft rejection (53). Collectively, these considerations underscore the need for autologous gene correction approaches to treat hypomorphic RAG diseases.

In conclusion, our work shows that MND-c.o.RAG1 LV mediated therapy allows partial B and T cell restoration in *Rag1^F971L/F971L^* and *Rag1^R972Q/R972Q^* hypomorphic mice, although counts remain lower than those of BMT WT mice. However, GT mice showed normalization of Ig production after *in vivo* challenges, suggesting that GT could provide a clinical benefit. Overt signs of autoimmunity were not present and there was no increase in autoantibody production, but it is important to consider that mice living in specific pathogen-free condition may not fully predict the outcome in humans. Therefore, it will be important to develop models with human cells to investigate efficacy and safety to support gene therapy for hypomorphic *RAG* diseases, where there clearly is an unmet clinical need for curative therapy.

## Data availability statement

The datasets presented in this study can be found in online repositories. The names of the repository/repositories and accession number(s) can be found below: PRJEB65467 (ENA).

## Ethics statement

The animal study was approved by Institutional Animal Care and Use Committee (IACUC) of San Raffaele Hospital. The study was conducted in accordance with the local legislation and institutional requirements.

## Author contributions

MC: Conceptualization, Formal Analysis, Investigation, Writing – review & editing. MD: Formal Analysis, Investigation, Writing – review & editing. ED: Investigation, Writing – review & editing. EF: Investigation, Writing – review & editing, Formal Analysis. SP: Formal Analysis, Investigation, Writing – review & editing. LS: Writing – review & editing, Formal Analysis, Investigation. AZ: Investigation, Writing – review & editing. DM: Investigation, Writing – review & editing. PU: Writing – review & editing, Formal Analysis. MZ: Investigation, Writing – review & editing, Formal Analysis. IG-F: Formal Analysis, Writing – review & editing, Investigation. AA: Investigation, Writing – review & editing. SI: Investigation, Writing – review & editing. LO: Investigation, Writing – review & editing. LN: Writing – review & editing, Methodology, Resources. KP-O: Writing – review & editing, Methodology, Resources. FS: Methodology, Resources, Writing – review & editing. AV: Resources, Writing – review & editing, Conceptualization, Funding acquisition, Supervision. VC: Writing – review & editing, Conceptualization, Formal Analysis, Investigation, Visualization, Writing – original draft.

## Supplementary material

Supplementary Figure 1 *In vitro* transduction. **(A).** Numbers of colony forming units (CFUs) obtained per 1000 cells on semi-solid medium to determine the clonogenic potential. **(B).** Vector copy number (VCN)/genome in bulk colonies. **(C).** VCN/genome in single CFUs. **(D).** Transduction efficiency, shown as percentage of transduced CFUs. WT, wild-type (n=8-23); F971L UT, untransduced *Rag1^F971L/F971L^* (n=1-11); F971L GT, gene therapy *Rag1^F971L/F971L^* (n=3-33); R972Q UT, untransduced *Rag1^R972Q/R972Q^* (n=4-20); R972Q GT, gene therapy *Rag1 ^R972Q/R972^* (n=6-67); n=8 independent experiments. Graphs show mean ± SD (panels **(A-B, D)** or median (panel **(C)**.

Supplementary Figure 2 Survival after transplant. **(A)** Survival of mice in n=4 experiments, terminated 6 months post transplant. **(B)** Survival of mice in n=2 experiments, terminated 12 months post transplant. F971L BMT WT, *Rag1^F971L/F971L^* transplanted with WT cells (n=4); R972Q BMT WT, *Rag1^R972Q/R972Q^* transplanted with WT cells (n=6 panel **(A)**, n=3 panel **(B)**; F971L BMT UT, *Rag1^F971L/F971L^* transplanted with untransduced cells (n=5 panel **(A)**, n=3 panel **(B)**; R972Q BMT UT, *Rag1^R972Q/R972Q^* transplanted with untransduced cells (n=7 panel **(A)**, n=3 panel B); F971L GT, *Rag1^F971L/F971L^* transplanted with gene therapy cells (n=19 panel **(A)**, n=4 panel **(B)**; R972Q GT, *Rag1^R972Q/R972Q^* transplanted with gene therapy cells (n=21 panel **(A)**, n=10 panel **(B)**.

Supplementary Figure 3 Immune subsets in peripheral blood. **(A, B).** Relative frequency of myeloid **(A)** and natural killer (NK) **(B)** cells were analyzed over time in *Rag1^F971L/F971L^* (square symbols) and *Rag1^R972Q/R972Q^* (round symbols). **(C, D).** Absolute counts of myeloid **(C)** and NK **(D)** cells in *Rag1^F971L/F971L^* (square symbols) and *Rag1^R972Q/R972Q^* (round symbols). **(E).** Total white blood cell (WBC) counts. WT ctrl, wild-type control (n=2-12); F971L ctrl, untreated *Rag1^F971L/F971L^* (n=1-8); R972Q ctrl, untreated *Rag1^R972Q/R972Q^* (n=1-8); F971L BMT WT, *Rag1^F971L/F971L^* transplanted with WT cells (n=2-6); R972Q BMT WT, *Rag1^R972Q/R972Q^* transplanted with WT cells (n=1-9); F971L BMT UT, *Rag1^F971L/F971L^* transplanted with untransduced cells (n=2-8); R972Q BMT UT, *Rag1^R972Q/R972Q^* transplanted with untransduced cells (n=1-8); F971L GT, *Rag1^F971L/F971L^* transplanted with gene therapy cells (n=4-23); R972Q GT, *Rag1^R972Q/R972Q^* transplanted with gene therapy cells (n=5-29). Graphs show mean ± standard deviation (SD). n=6 independent experiments. Statistical analysis: non parametric one-way ANOVA, * p<0.05, ** p<0.01, *** p<0.001, **** p<0.0001, ns not significant p>0.05; in red are shown differences between GT and mutated *Rag1* controls, in grey between GT and BMT WT.

Supplementary Figure 4 B cell reconstitution in bone marrow 6 months post-transplant. **(A-E)**. Frequency of B cell subsets in bone marrow (BM) of treated and control (ctrl) mice: pre-proB **(A)**, proB **(B)**, preB **(C)**, immature **(D)** and mature recirculating B cells **(E)**. **(F-J)**. Absolute counts of B cell subsets in bone marrow (BM) of treated and control (ctrl) mice: pre-proB **(F)**, proB **(G)**, preB **(H)**, immature **(I)** and mature recirculating B cells **(J)**. WT ctrl, wild-type control (n=15); F971L ctrl, untreated *Rag1^F971L/F971L^* (n=7); R972Q ctrl, untreated *Rag1^R972Q/R972Q^* (n=11); F971L BMT WT, *Rag1^F971L/F971L^* transplanted with WT cells (n=4); R972Q BMT WT, *Rag1^R972Q/R972Q^* transplanted with WT cells (n=6); F971L BMT UT, *Rag1^F971L/F971L^* transplanted with untransduced cells (n=5); R972Q BMT UT, *Rag1^R972Q/R972Q^* transplanted with untransduced cells (n=5); F971L GT, *Rag1^F971L/F971L^* transplanted with gene therapy cells (n=16); R972Q GT, *Rag1^R972Q/R972Q^* transplanted with gene therapy cells (n=19). n=4 independent experiments. Graphs show mean ± SD. Statistical analysis: non parametric one-way ANOVA, * p<0.05, ** p<0.01, ns, not significant p>0.05.

Supplementary Figure 5 B cell reconstitution in spleen 6 months post-transplant. **(A-D)**. Absolute counts of B **(A)**, T **(B)**, myeloid **(C)** and natural killer (NK) cells **(D)** in the spleen of control and treated mice. **(E-H).** Frequency of marginal zone **(E)**, follicular **(F)**, transitional 1 **(G)** and transitional 2 **(H)** B cells of the spleen. **(I)**. Fraction of white pulp area of the spleen. **J.** Immunoglobulin A (IgA) levels in plasma. Dotted lines indicate the range of IgA levels in untreated age-matched WT mice. **K.** Total immunoglobulin G (IgG) levels (Ig1, IgG2a, IgG2b, and IgG3) in plasma. Dotted lines indicate the range of IgG levels in untreated age-matched WT mice. WT ctrl, wild-type control (n=7-18); F971L ctrl, untreated *Rag1^F971L/F971L^* (n=6-7); R972Q ctrl, untreated *Rag1^R972Q/R972Q^* (n=3-15); F971L BMT WT, *Rag1^F971L/F971L^* transplanted with WT cells (n=5-8); R972Q BMT WT, *Rag1^R972Q/R972Q^* transplanted with WT cells (n=2-9); F971L BMT UT, *Rag1^F971L/F971L^* transplanted with untransduced cells (n=5-8); R972Q BMT UT, *Rag1^R972Q/R972Q^* transplanted with untransduced cells (n=2-7); F971L GT, *Rag1^F971L/F971L^* transplanted with gene therapy cells (n=15-20); R972Q GT, *Rag1^R972Q/R972Q^* transplanted with gene therapy cells (n=4-29). Graphs show mean ± standard deviation (SD). Statistical analysis: non parametric one-way ANOVA: * p<0.05, ns, not significant p>0.05. n=4 (panels A-H, J-K) or n=2 (panel I) independent experiments.

Supplementary Figure 6 T cell reconstitution at 6 months post-transplant. **(A-D)**. Frequency of double negative (DN) stages of thymocytes: DN1 **(A)**, DN2 **(B)**, DN3 **(C)**, DN4 **(D)**. **(E-G).** Frequency of naïve **(E)**, effector **(F)** and memory **(G)** T cells of the spleen. WT ctrl, wild-type control (n=15); F971L ctrl, untreated *Rag1^F971L/F971L^* (n=7); R972Q ctrl, untreated *Rag1^R972Q/R972Q^* (n=11); F971L BMT WT, *Rag1^F971L/F971L^* transplanted with WT cells (n=4); R972Q BMT WT, *Rag1^R972Q/R972Q^* transplanted with WT cells (n=6); F971L BMT UT, *Rag1^F971L/F971L^* transplanted with untransduced cells (n=5); R972Q BMT UT, *Rag1^R972Q/R972Q^* transplanted with untransduced cells (n=5); F971L GT, *Rag1^F971L/F971L^* transplanted with gene therapy cells (n=16); R972Q GT, *Rag1^R972Q/R972Q^* transplanted with gene therapy cells (n=19). n=4 independent experiments. Graphs show mean ± SD. Statistical analysis: non parametric one-way ANOVA: * p<0.05, ** p<0.01, ns not significant p>0.05.

Supplementary Figure 7 Thymic reconstitution at 6 months post-transplant. **(A).** Representative pictures of hematoxilyn and eosin staining of thymic tissue sections. **(B).** Representative pictures of cytokeratin 5 (CK5) and CD3 stainings of thymic tissue sections. **(C)**. Correlation between vector copy number/genome (VCN) in thymus and medulla/cortex (M/C) ratio. Spearman r value is reported. F971L/R972Q BMT WT, *Rag1^F971L/F971L^* or *Rag1^R972Q/R972Q^* transplanted with WT cells; F971L/R972Q BMT UT, *Rag1^F971L/F971L^* or *Rag1^R972Q/R972Q^* transplanted with untransduced cells; F971L/R972Q GT, *Rag1^F971L/F971L^* or *Rag1^R972Q/R972Q^* transplanted with gene therapy cells. Statistical analysis: Spearman correlation (panel C), * p<0.05. n=3 independent experiments.

Supplementary Figure 8 Immune cell reconstitution in bone marrow at termination 12 months post-transplant. **(A-F).** Absolute counts of B cell subsets **(A)** in bone marrow (BM) of treated and control (ctrl) mice: pre-pro B **(B)**, proB **(C)**, preB **(D)**, immature **(E)** and mature B cells **(F)**. **G-K.** Absolute counts of immune subsets **(G)** in the BM: B **(H)**, T **(I)**, myeloid **(J)** and natural killer (NK) **(K)** cells. **L.** Vector copy number (VCN)/genome in single cell suspensions from bone marrow of hypomorphic gene therapy (GT) mice. WT ctrl, wild-type control (n=5); F971L ctrl, untreated *Rag1^F971L/F971L^* (n=1); R972Q ctrl, untreated *Rag1^R972Q/R972Q^* (n=5); F971L BMT WT, *Rag1^F971L/F971L^* transplanted with WT cells (n=4); R972Q BMT WT, *Rag1^R972Q/R972Q^* transplanted with WT cells (n=3); F971L BMT UT, *Rag1^F971L/F971L^* transplanted with untransduced cells (n=2); R972Q BMT UT, *Rag1^R972Q/R972Q^* transplanted with untransduced cells (n=1); F971L GT, *Rag1^F971L/F971L^* transplanted with gene therapy cells (n=3-4); R972Q GT, *Rag1^R972Q/R972Q^* transplanted with gene therapy cells (n=8-9). Graphs show mean ± standard deviation (SD). n=2 independent experiments. Statistical analysis (panels **(B-F, H-K)**: non parametric one-way ANOVA: ns not significant p>0.05.

Supplementary Figure 9 Immune cell reconstitution in thymus at termination 12 months post-transplant. **(A-E).** Frequency of double negative (DN) stages **(A)** of thymocytes: DN1 **(B)**, DN2 **(C)**, DN3 **(D)**, DN4 **(E)**. **(F).** Vector copy number (VCN)/genome in single cell suspensions thymus of hypomorphic gene therapy (GT) mice. WT ctrl, wild-type control (n=5); F971L ctrl, untreated *Rag1^F971L/F971L^* (n=1); R972Q ctrl, untreated *Rag1^R972Q/R972Q^* (n=5); F971L BMT WT, *Rag1^F971L/F971L^* transplanted with WT cells (n=4); R972Q BMT WT, *Rag1^R972Q/R972Q^* transplanted with WT cells (n=3); F971L BMT UT, *Rag1^F971L/F971L^* transplanted with untransduced cells (n=2); R972Q BMT UT, *Rag1^R972Q/R972Q^* transplanted with untransduced cells (n=1); F971L GT, *Rag1^F971L/F971L^* transplanted with gene therapy cells (n=3-4); R972Q GT, *Rag1^R972Q/R972Q^* transplanted with gene therapy cells (n=8-9). Graphs show mean ± standard deviation (SD). n=2. Statistical analysis (panels B-E): non parametric one-way ANOVA: * p<0.05, ns not significant p>0.05.

Supplementary Figure 10 Immune cell reconstitution in spleen at termination 12 months post-transplant. **(A-E).** Absolute counts of immune subsets **(A)** in the spleen: B **(B)**, T **(C)**, myeloid **(D)** and natural killer (NK) **(E)** cells in the spleen. **(F).** Vector copy number (VCN)/genome in single cell suspensions from spleen of hypomorphic gene therapy (GT) mice. **(G).** V- and J-gene recombination of T cell receptors (TCR) in splenocytes. Each bow between V- and J-gene represents a clonotype. The broader a bow, the higher relative frequency was detected for the respective clonotype. WT ctrl, wild-type control (n=5); F971L ctrl, untreated *Rag1^F971L/F971L^* (n=1); R972Q ctrl, untreated *Rag1^R972Q/R972Q^* (n=5); F971L BMT WT, *Rag1^F971L/F971L^* transplanted with WT cells (n=4); R972Q BMT WT, *Rag1^R972Q/R972Q^* transplanted with WT cells (n=3); F971L BMT UT, *Rag1^F971L/F971L^* transplanted with untransduced cells (n=2); R972Q BMT UT, *Rag1^R972Q/R972Q^* transplanted with untransduced cells (n=1); F971L GT, *Rag1^F971L/F971L^* transplanted with gene therapy cells (n=3-4); R972Q GT, *Rag1^R972Q/R972Q^* transplanted with gene therapy cells (n=8-9). Graphs show mean ± standard deviation (SD). n=2 (panels A-F) or n=1 (panel G) independent experiments. Statistical analysis (panels B-E): non parametric one-way ANOVA: * p<0.05, ns not significant p>0.05.

Supplementary Figure 11 Donor chimerism in CD45-mismatched transplants, terminated 6 months post-transplant. **(A-C).** Chimerism of donor cells (CD45.1) over time in the B **(A)**, myeloid **(B)** and NK **(C)** cells of peripheral blood. **(D-F)**. Frequency of T cells subsets in donor CD45.1 cells of the spleen: naïve **(D)**, effector **(E)** and memory **(F)** T cells. **G-I**. Frequency of T cells subsets in recipient CD45.2 cells of the spleen: naïve **(G)**, effector **(H)** and memory **(I)** T cells. **J-N.** Chimerism of CD45.1+ donor cells in the pre-proB **(J)**, proB **(K)**, preB **(L)**, immature **(M)** and mature recirculating B **(N)** cells of the bone marrow. **(O).** Chimerism of CD45.1+ donor cells in the Lin- Sca1+ cKit+ progenitor cells of the bone marrow. **(P-R).** Chimerism of donor cells (CD45.1) in the B **(P)**, myeloid **(Q)** and NK **(R)** cells of the spleen. R972Q BMT WT, *Rag1^R972Q/R972Q^* transplanted with WT cells (n=4); R972Q BMT UT, *Rag1^R972Q/R972Q^* transplanted with untransduced cells (n=3); R972Q GT, *Rag1^R972Q/R972Q^* transplanted with gene therapy cells (n=15). n=2 independent experiments. Graphs show mean ± SD (panels A-I) or median (panels J-R). Statistical analysis: non parametric one-way ANOVA, * p<0.05, ** p<0.01, *** p<0.001, ns not significant p>0.05. In panels J-R, only statistically significant comparisons are shown.

Supplementary Figure 12 Short-term donor chimerism in CD45-mismatched transplants, terminated 3 weeks post-transplant. **(A-C).** Chimerism of donor cells (CD45.1) in the total T **(A)**, CD4+ T **(B)** and CD8+ T **(C)** cells of peripheral blood. **(D-G).** Chimerism of donor cells in the double negative (DN, **(D)**, double positive (DP, **(E)**, single positive 4 (SP4, **(F)** and single positive 8 (SP8, **(G)** subsets of the thymus. Graphs show frequency of donor CD45.1 cells (top) or fraction of donor CD45.1+ and recipient CD45.2+ cells (bottom). **(H).** Vector copy number (VCN)/genome in single cell suspensions from the thymus of hypomorphic gene therapy (GT) mice. **(I)**. Frequency of donor CD45.1 cells in the T cells of the spleen. **(J).** Vector copy number (VCN)/genome in single cell suspensions from spleen of hypomorphic gene therapy (GT) mice. **(K-N).** Distribution of CD4- CD8- **(K)**, CD4+ **(L)**, CD8+ **(M)** T cells **(N)** of the spleen. WT ctrl, wild-type control (n=2); F971L ctrl, untreated *Rag1^F971L/F971L^* (n=1); R972Q ctrl, untreated *Rag1^R972Q/R972Q^* (n=1); F971L BMT WT, *Rag1^F971L/F971L^* transplanted with WT cells (n=2); R972Q BMT WT, *Rag1^R972Q/R972Q^* transplanted with WT cells (n=4); F971L BMT UT, *Rag1^F971L/F971L^* transplanted with untransduced cells (n=1); R972Q BMT UT, *Rag1^R972Q/R972Q^* transplanted with untransduced cells (n=4); F971L GT, *Rag1^F971L/F971L^* transplanted with gene therapy cells (n=4); R972Q GT, *Rag1^R972Q/R972Q^* transplanted with gene therapy cells (n=7). Graphs show mean ± standard deviation (SD). n=2 independent experiments. Statistical analysis: non parametric one-way ANOVA, * p<0.05, ** p<0.01, ns not significant p>0.05. In panels A-G and I, only statistically significant comparisons are shown; in panels K-M, all performed comparisons are shown.

Supplementary Figure 13 Integration site analysis. **(A, B).** Cumulative retrieval frequencies of the ten most prominent integration sites (ISs) detected in the thymus and spleen of GT animals with abnormal **(A)** or normal **(B)** thymic tissue. Vector copy numbers/genome (VCNs) of thymus (Thy) and spleen (Spl) are shown. Sequence data from all S-EPTS/LM-PCR (shearing extension primer tag selection ligation-mediated PCR) amplicons are combined. Sequence count of the ten most prominent ISs (colour) and sequence count of all remaining ISs (grey) as well as total sequence count from all amplicons are shown at the bottom of the table. RefSeq names of genes located closest to the respective IS are given in the table. Relative sequence count contributions of the ten most prominent ISs and all remaining mappable IS are shown (frequency).
