# Supplementary figures and images for: Partial correction of immunodeficiency by lentiviral vector gene therapy in mouse models carrying Rag1 hypomorphic mutations
Source: Front Immunol. 2023 Nov 13;14:1268620. doi: 10.3389/fimmu.2023.1268620 (PMC10679457; doi:10.3389/fimmu.2023.1268620)

Supplementary figure S1

**A**

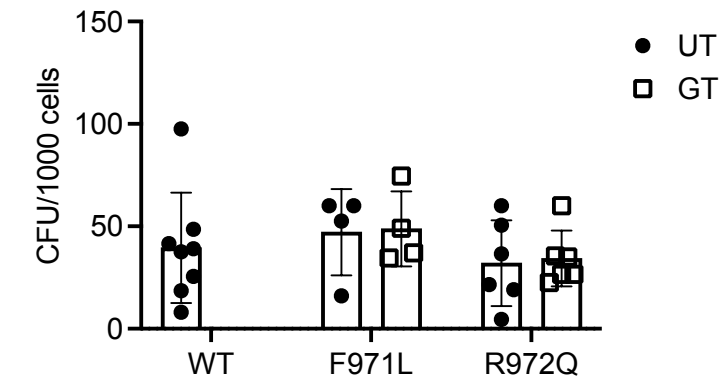

**B**

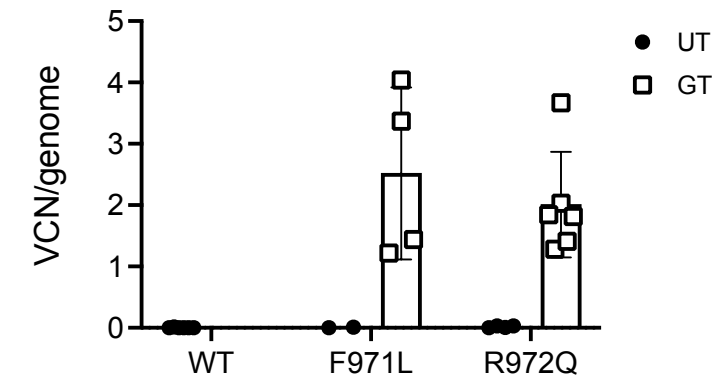

**C**

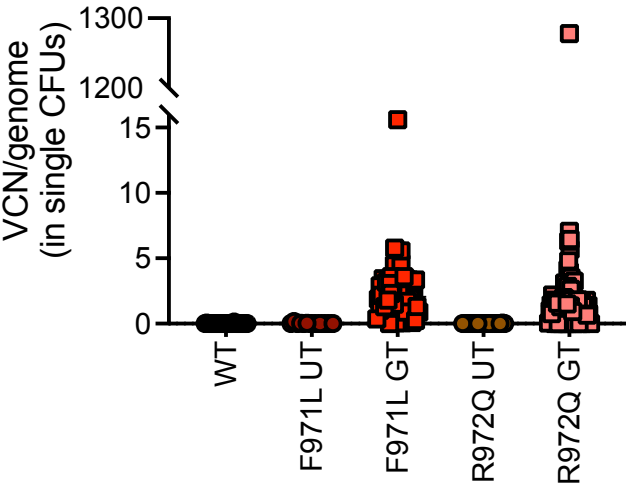

**D**

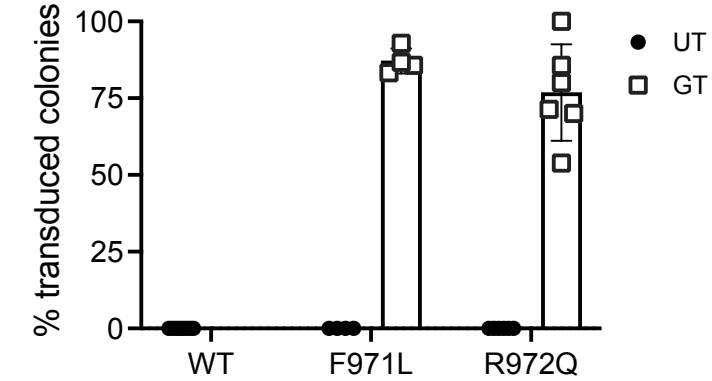

Supplement: Supplementary Figure 1 — In vitro transduction. (A). Numbers of colony forming units (CFUs) obtained per 1000 cells on semi-solid medium to determine the clonogenic potential. (B). Vector copy number (VCN)/genome in bulk colonies. (C). VCN/genome in single CFUs. (D). Transduction efficiency, shown as percentage of transduced CFUs. WT, wild-type (n=8-23); F971L UT, untransduced Rag1F971L/F971L (n=1-11); F971L GT, gene therapy Rag1F971L/F971L (n=3-33); R972Q UT, untransduced Rag1R972Q/R972Q (n=4-20); R972Q GT, gene therapy Rag1 R972Q/R972 (n=6-67); n=8 independent experiments. Graphs show mean ± SD (panels (A-B, D) or median (panel (C). [file Image_1.pdf]

Supplementary figure S2

A

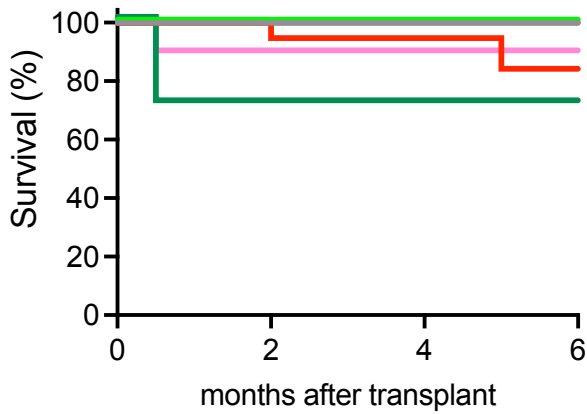

B

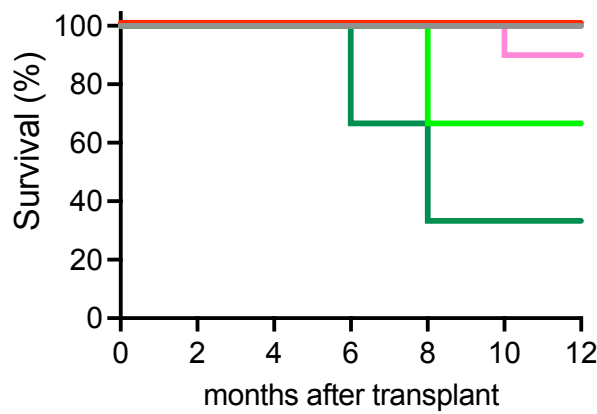

Supplement: Supplementary Figure 2 — Survival after transplant. (A) Survival of mice in n=4 experiments, terminated 6 months post transplant. (B) Survival of mice in n=2 experiments, terminated 12 months post transplant. F971L BMT WT, Rag1F971L/F971L transplanted with WT cells (n=4); R972Q BMT WT, Rag1R972Q/R972Q transplanted with WT cells (n=6 panel (A), n=3 panel (B); F971L BMT UT, Rag1F971L/F971L transplanted with untransduced cells (n=5 panel (A), n=3 panel (B); R972Q BMT UT, Rag1R972Q/R972Q transplanted with untransduced cells (n=7 panel (A), n=3 panel B); F971L GT, Rag1F971L/F971L transplanted with gene therapy cells (n=19 panel (A), n=4 panel (B); R972Q GT, Rag1R972Q/R972Q transplanted with gene therapy cells (n=21 panel (A), n=10 panel (B). [file Image_2.pdf]

# Supplementary figure S3

**A**

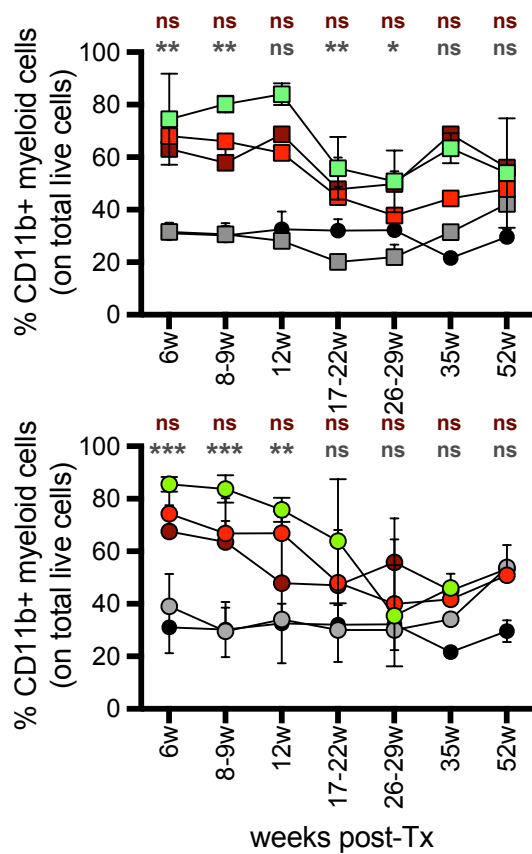

**B**

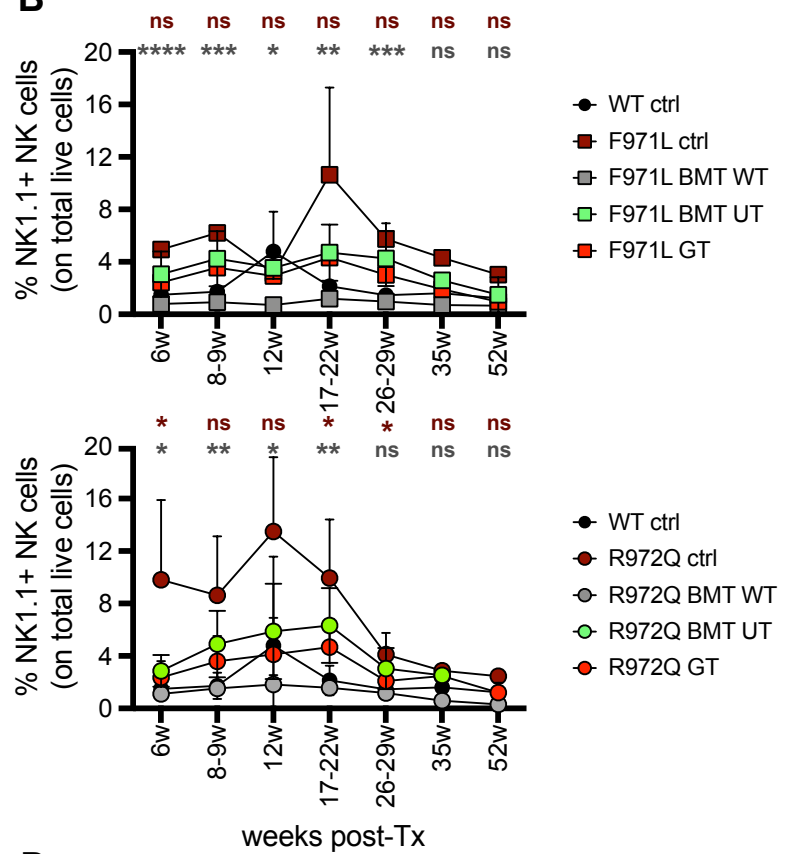

**C**

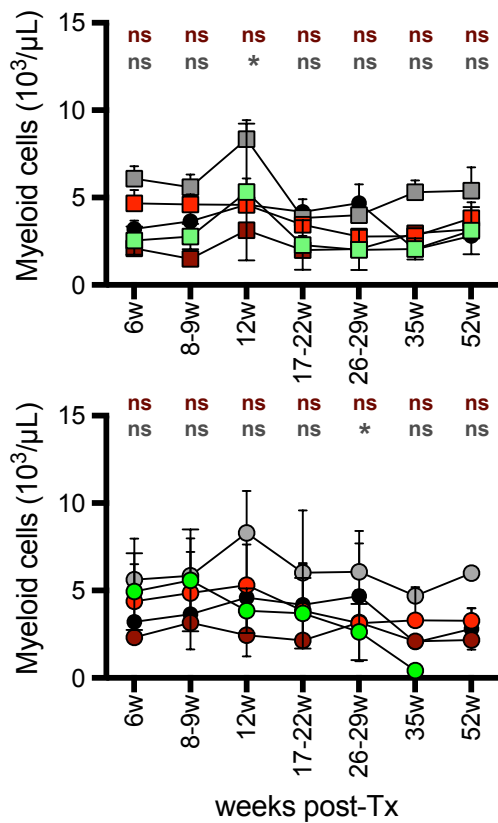

**D**

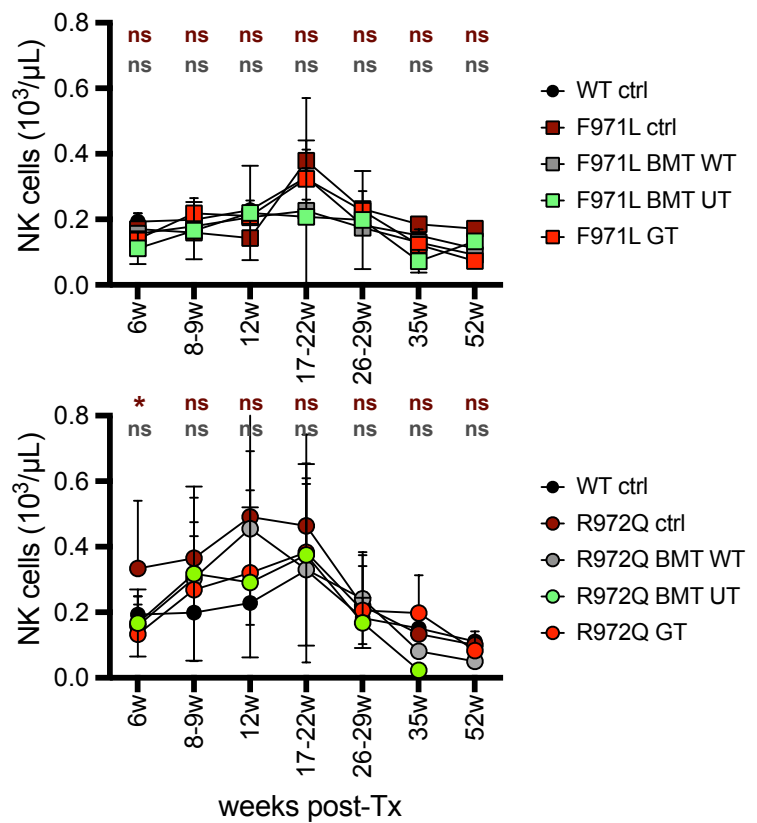

**E**

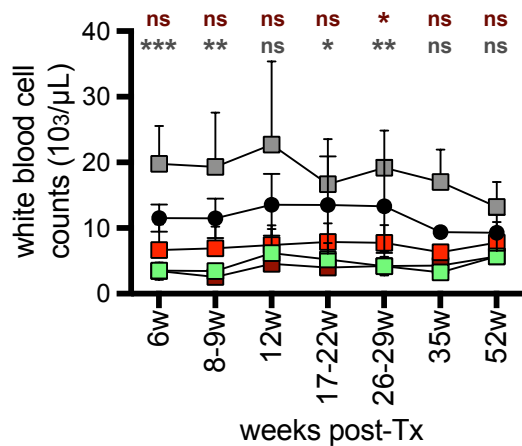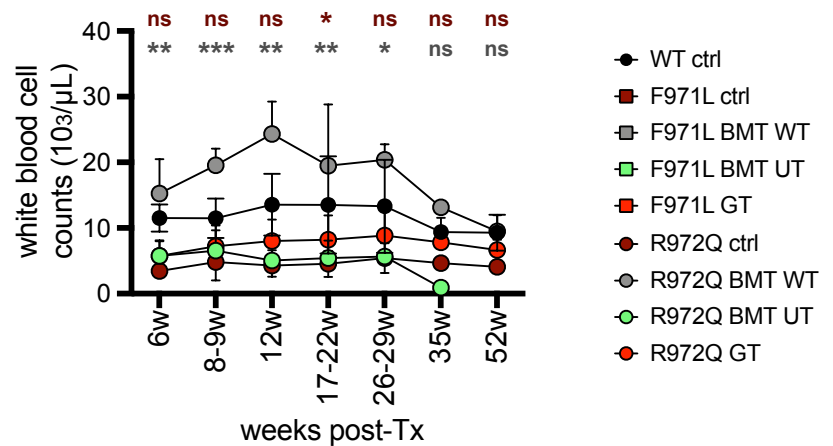

Supplement: Supplementary Figure 3 — Immune subsets in peripheral blood. (A, B). Relative frequency of myeloid (A) and natural killer (NK) (B) cells were analyzed over time in Rag1F971L/F971L (square symbols) and Rag1R972Q/R972Q (round symbols). (C, D). Absolute counts of myeloid (C) and NK (D) cells in Rag1F971L/F971L (square symbols) and Rag1R972Q/R972Q (round symbols). (E). Total white blood cell (WBC) counts. WT ctrl, wild-type control (n=2-12); F971L ctrl, untreated Rag1F971L/F971L (n=1-8); R972Q ctrl, untreated Rag1R972Q/R972Q (n=1-8); F971L BMT WT, Rag1F971L/F971L transplanted with WT cells (n=2-6); R972Q BMT WT, Rag1R972Q/R972Q transplanted with WT cells (n=1-9); F971L BMT UT, Rag1F971L/F971L transplanted with untransduced cells (n=2-8); R972Q BMT UT, Rag1R972Q/R972Q transplanted with untransduced cells (n=1-8); F971L GT, Rag1F971L/F971L transplanted with gene therapy cells (n=4-23); R972Q GT, Rag1R972Q/R972Q transplanted with gene therapy cells (n=5-29). Graphs show mean ± standard deviation (SD). n=6 independent experiments. Statistical analysis: non parametric one-way ANOVA, * p<0.05, ** p<0.01, *** p<0.001, **** p<0.0001, ns not significant p>0.05; in red are shown differences between GT and mutated Rag1 controls, in grey between GT and BMT WT. [file Image_3.pdf]

Supplementary Figure S4

**A**

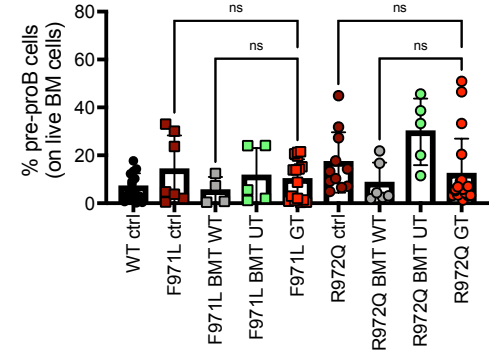

**B**

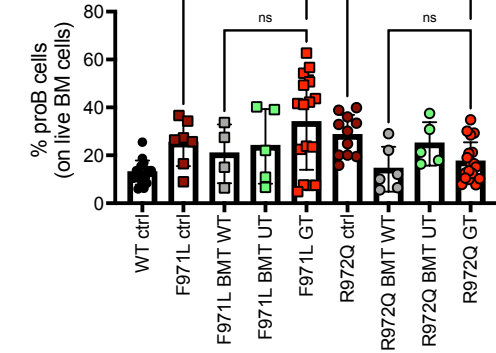

**C**

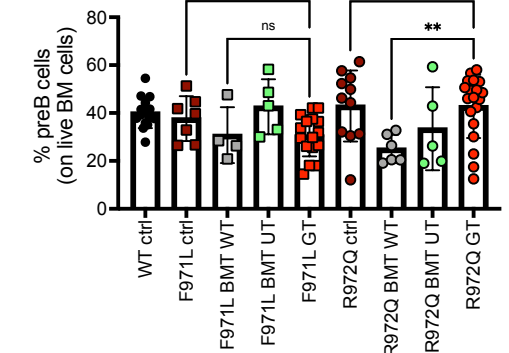

**D**

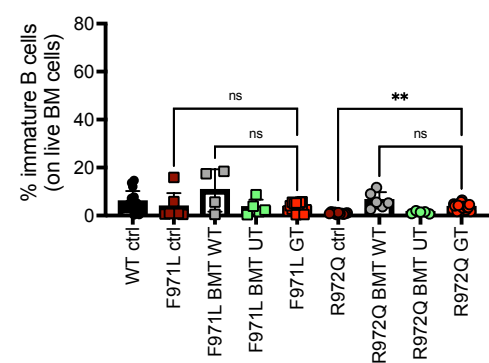

**E**

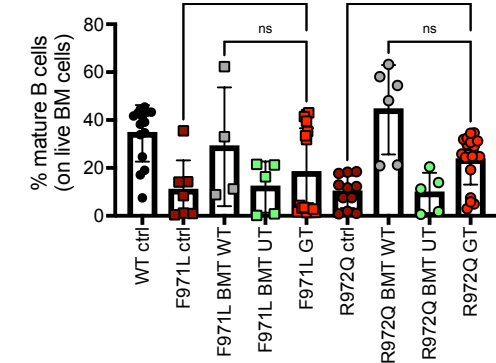

**F**

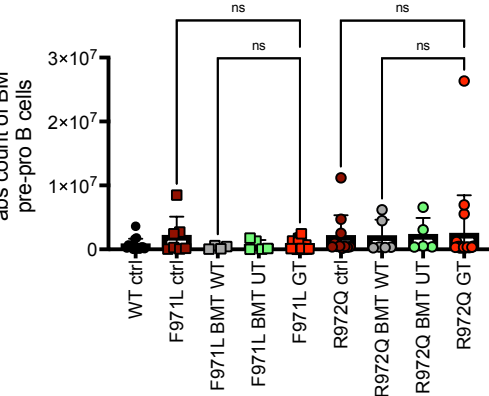

**G**

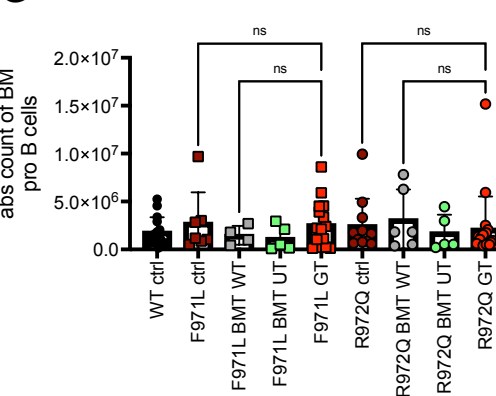

**H**

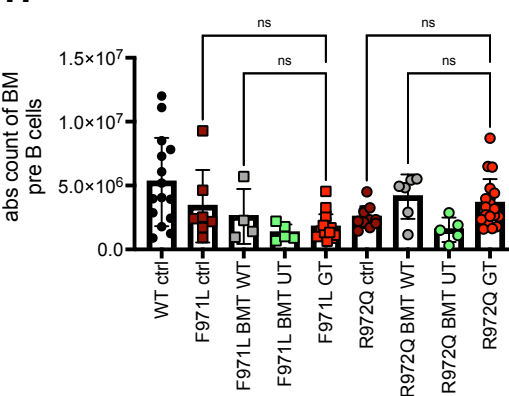

**I**

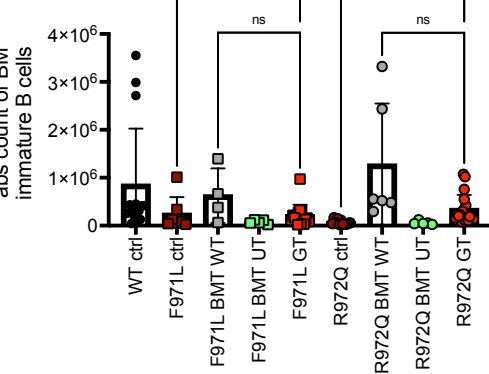

**J**

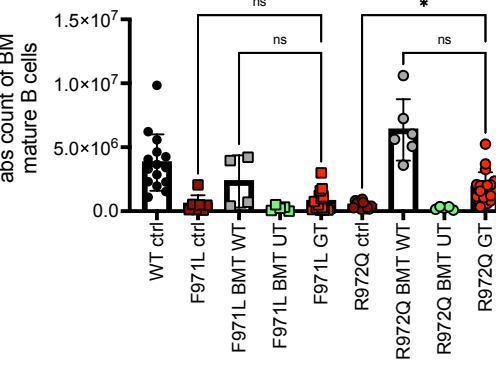

Supplement: Supplementary Figure 4 — B cell reconstitution in bone marrow 6 months post-transplant. (A-E). Frequency of B cell subsets in bone marrow (BM) of treated and control (ctrl) mice: pre-proB (A), proB (B), preB (C), immature (D) and mature recirculating B cells (E). (F-J). Absolute counts of B cell subsets in bone marrow (BM) of treated and control (ctrl) mice: pre-proB (F), proB (G), preB (H), immature (I) and mature recirculating B cells (J). WT ctrl, wild-type control (n=15); F971L ctrl, untreated Rag1F971L/F971L (n=7); R972Q ctrl, untreated Rag1R972Q/R972Q (n=11); F971L BMT WT, Rag1F971L/F971L transplanted with WT cells (n=4); R972Q BMT WT, Rag1R972Q/R972Q transplanted with WT cells (n=6); F971L BMT UT, Rag1F971L/F971L transplanted with untransduced cells (n=5); R972Q BMT UT, Rag1R972Q/R972Q transplanted with untransduced cells (n=5); F971L GT, Rag1F971L/F971L transplanted with gene therapy cells (n=16); R972Q GT, Rag1R972Q/R972Q transplanted with gene therapy cells (n=19). n=4 independent experiments. Graphs show mean ± SD. Statistical analysis: non parametric one-way ANOVA, * p<0.05, ** p<0.01, ns, not significant p>0.05. [file Image_4.pdf]

# Supplementary figure S5

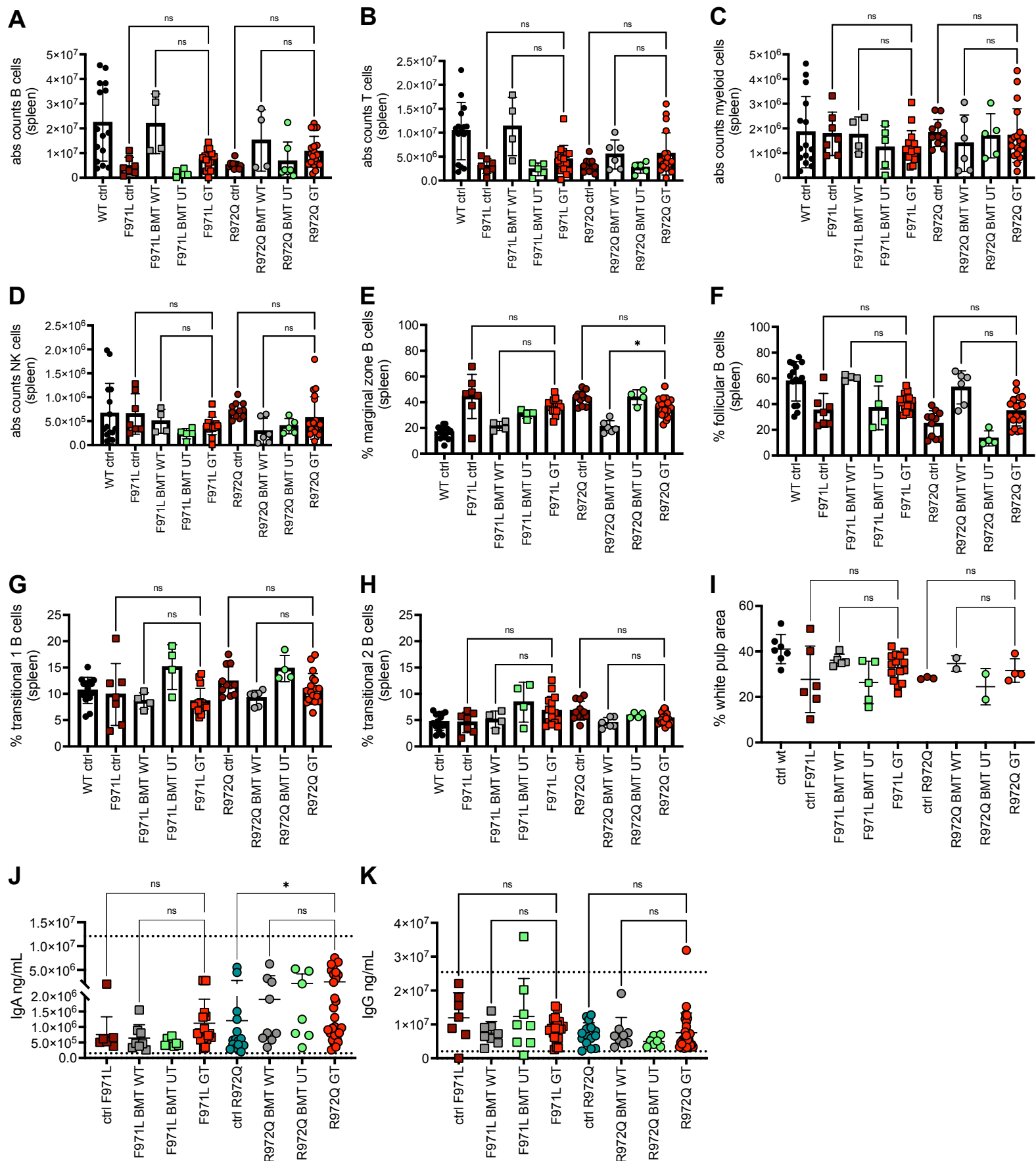

Supplement: Supplementary Figure 5 — B cell reconstitution in spleen 6 months post-transplant. (A-D). Absolute counts of B (A), T (B), myeloid (C) and natural killer (NK) cells (D) in the spleen of control and treated mice. (E-H). Frequency of marginal zone (E), follicular (F), transitional 1 (G) and transitional 2 (H) B cells of the spleen. (I). Fraction of white pulp area of the spleen. J. Immunoglobulin A (IgA) levels in plasma. Dotted lines indicate the range of IgA levels in untreated age-matched WT mice. K. Total immunoglobulin G (IgG) levels (Ig1, IgG2a, IgG2b, and IgG3) in plasma. Dotted lines indicate the range of IgG levels in untreated age-matched WT mice. WT ctrl, wild-type control (n=7-18); F971L ctrl, untreated Rag1F971L/F971L (n=6-7); R972Q ctrl, untreated Rag1R972Q/R972Q (n=3-15); F971L BMT WT, Rag1F971L/F971L transplanted with WT cells (n=5-8); R972Q BMT WT, Rag1R972Q/R972Q transplanted with WT cells (n=2-9); F971L BMT UT, Rag1F971L/F971L transplanted with untransduced cells (n=5-8); R972Q BMT UT, Rag1R972Q/R972Q transplanted with untransduced cells (n=2-7); F971L GT, Rag1F971L/F971L transplanted with gene therapy cells (n=15-20); R972Q GT, Rag1R972Q/R972Q transplanted with gene therapy cells (n=4-29). Graphs show mean ± standard deviation (SD). Statistical analysis: non parametric one-way ANOVA: * p<0.05, ns, not significant p>0.05. n=4 (panels A-H, J-K) or n=2 (panel I) independent experiments. [file Image_5.pdf]

# Supplementary figure S6

**A**

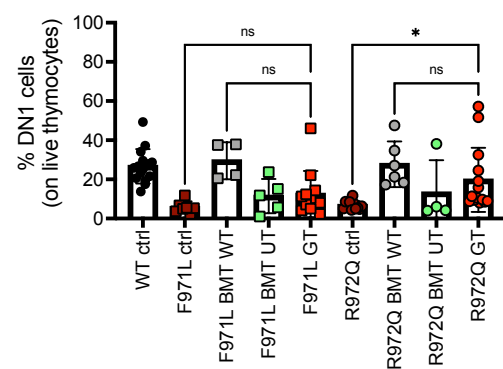

**B**

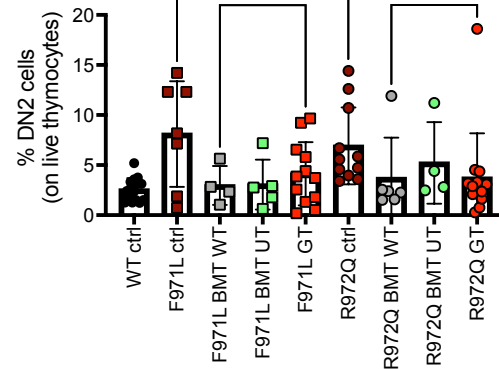

**C**

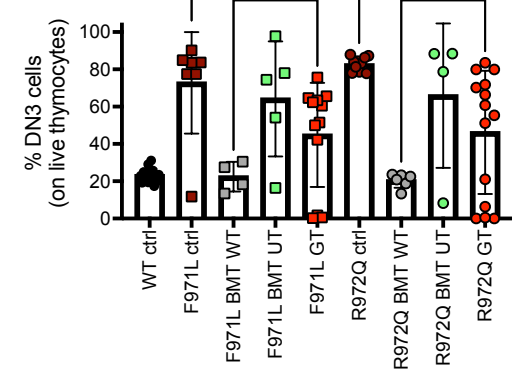

**D**

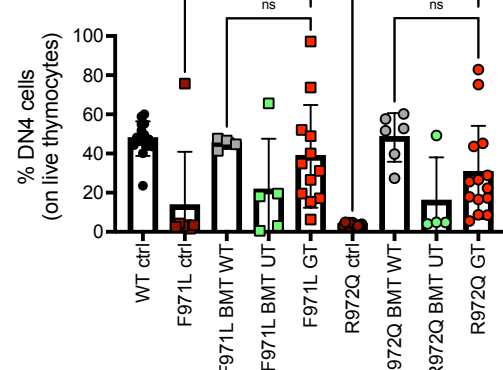

**E**

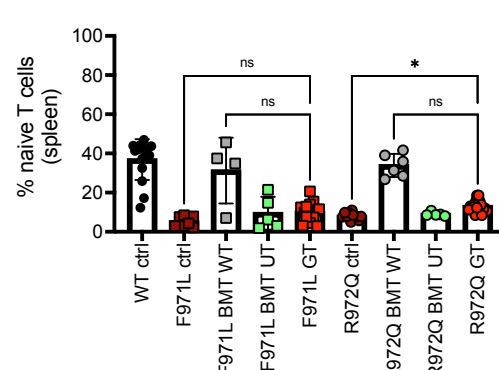

**F**

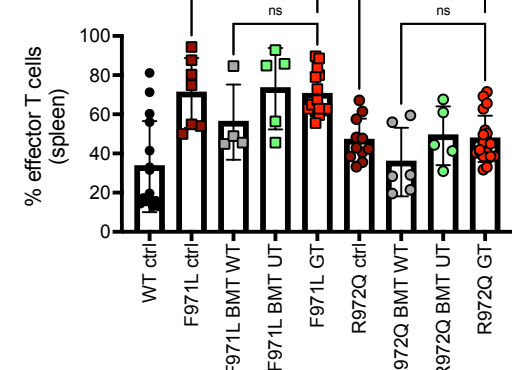

**G**

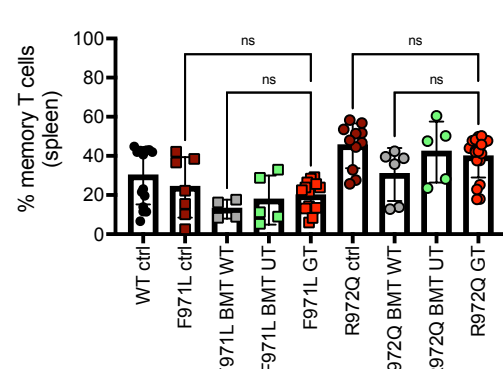

Supplement: Supplementary Figure 6 — T cell reconstitution at 6 months post-transplant. (A-D). Frequency of double negative (DN) stages of thymocytes: DN1 (A), DN2 (B), DN3 (C), DN4 (D). (E-G). Frequency of naïve (E), effector (F) and memory (G) T cells of the spleen. WT ctrl, wild-type control (n=15); F971L ctrl, untreated Rag1F971L/F971L (n=7); R972Q ctrl, untreated Rag1R972Q/R972Q (n=11); F971L BMT WT, Rag1F971L/F971L transplanted with WT cells (n=4); R972Q BMT WT, Rag1R972Q/R972Q transplanted with WT cells (n=6); F971L BMT UT, Rag1F971L/F971L transplanted with untransduced cells (n=5); R972Q BMT UT, Rag1R972Q/R972Q transplanted with untransduced cells (n=5); F971L GT, Rag1F971L/F971L transplanted with gene therapy cells (n=16); R972Q GT, Rag1R972Q/R972Q transplanted with gene therapy cells (n=19). n=4 independent experiments. Graphs show mean ± SD. Statistical analysis: non parametric one-way ANOVA: * p<0.05, ** p<0.01, ns not significant p>0.05. [file Image_6.pdf]

# Supplementary figure S7

**A**

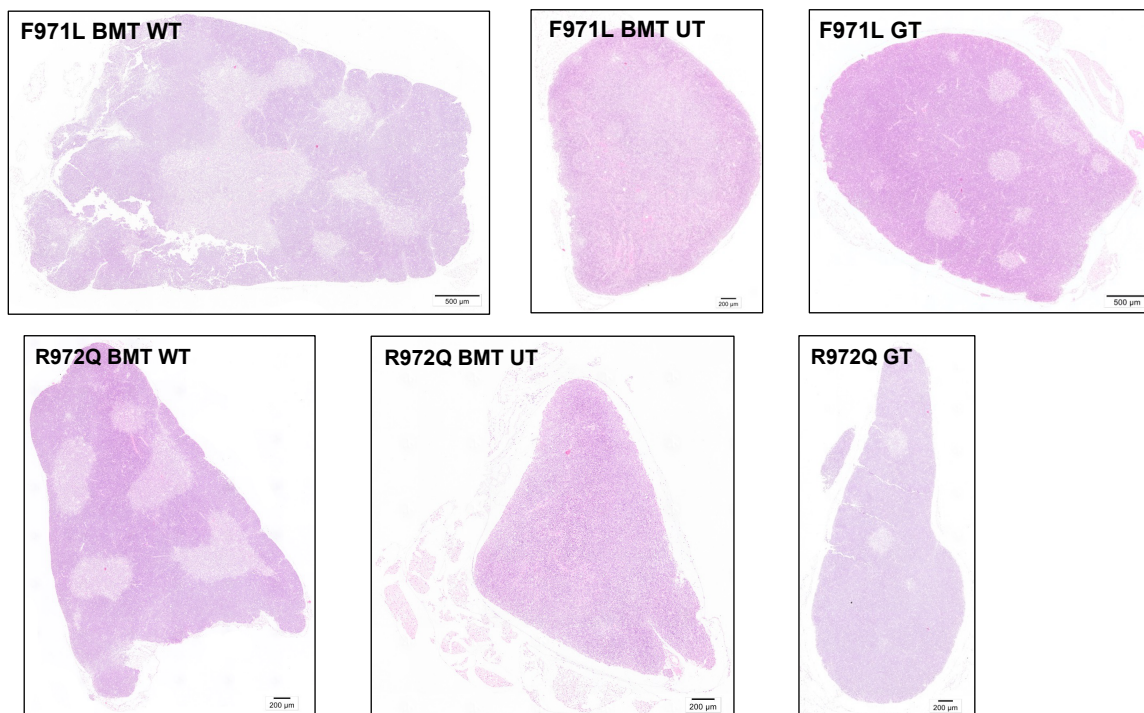

**B**

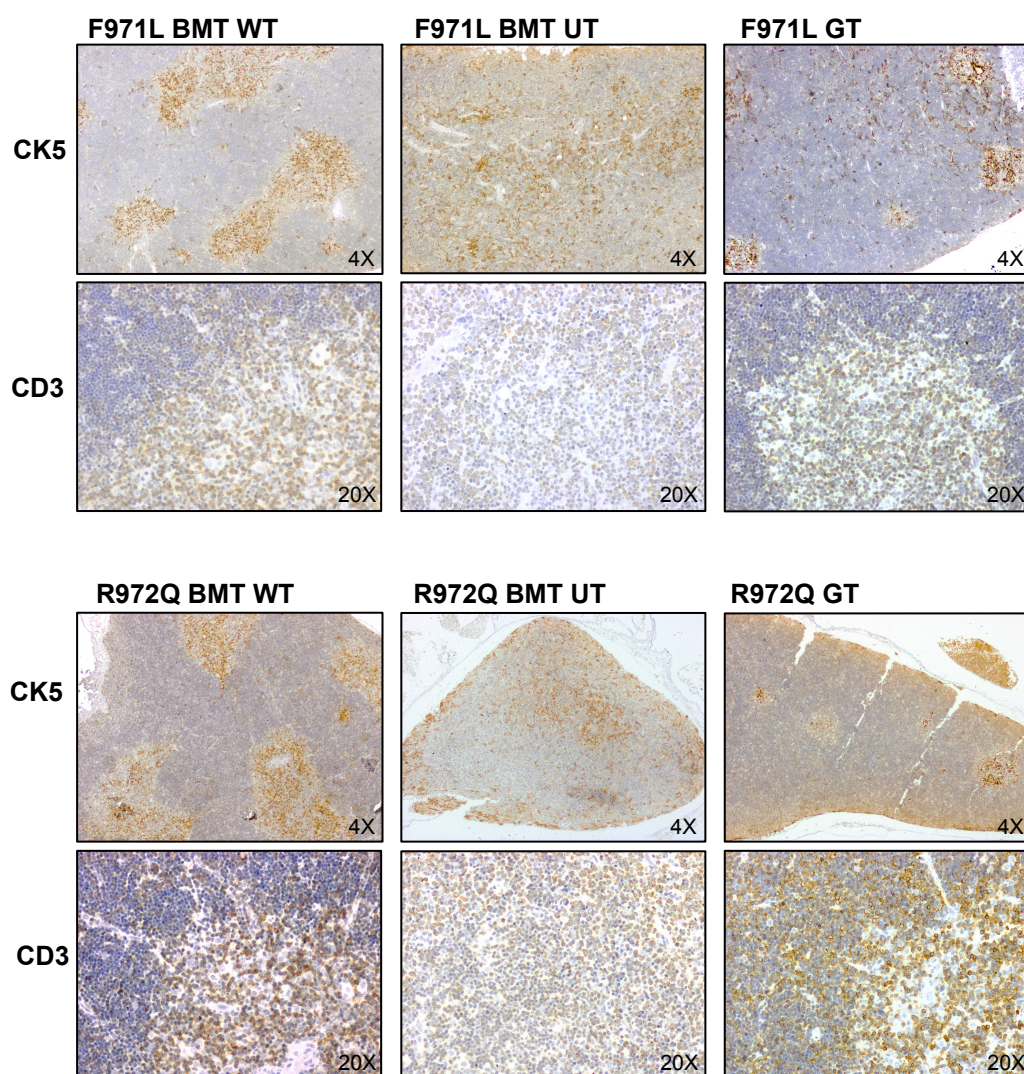

**C**

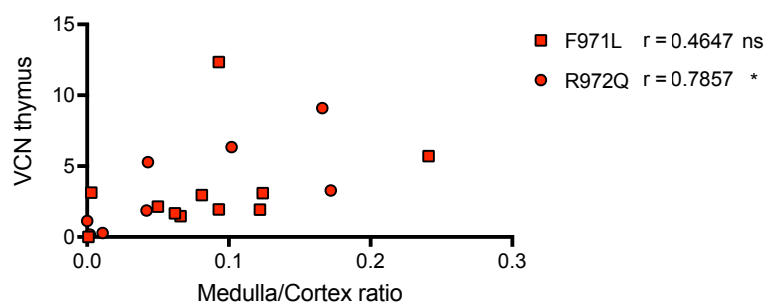

Supplement: Supplementary Figure 7 — Thymic reconstitution at 6 months post-transplant. (A). Representative pictures of hematoxilyn and eosin staining of thymic tissue sections. (B). Representative pictures of cytokeratin 5 (CK5) and CD3 stainings of thymic tissue sections. (C). Correlation between vector copy number/genome (VCN) in thymus and medulla/cortex (M/C) ratio. Spearman r value is reported. F971L/R972Q BMT WT, Rag1F971L/F971L or Rag1R972Q/R972Q transplanted with WT cells; F971L/R972Q BMT UT, Rag1F971L/F971L or Rag1R972Q/R972Q transplanted with untransduced cells; F971L/R972Q GT, Rag1F971L/F971L or Rag1R972Q/R972Q transplanted with gene therapy cells. Statistical analysis: Spearman correlation (panel C), * p<0.05. n=3 independent experiments. [file Image_7.pdf]

# Supplementary figure S8

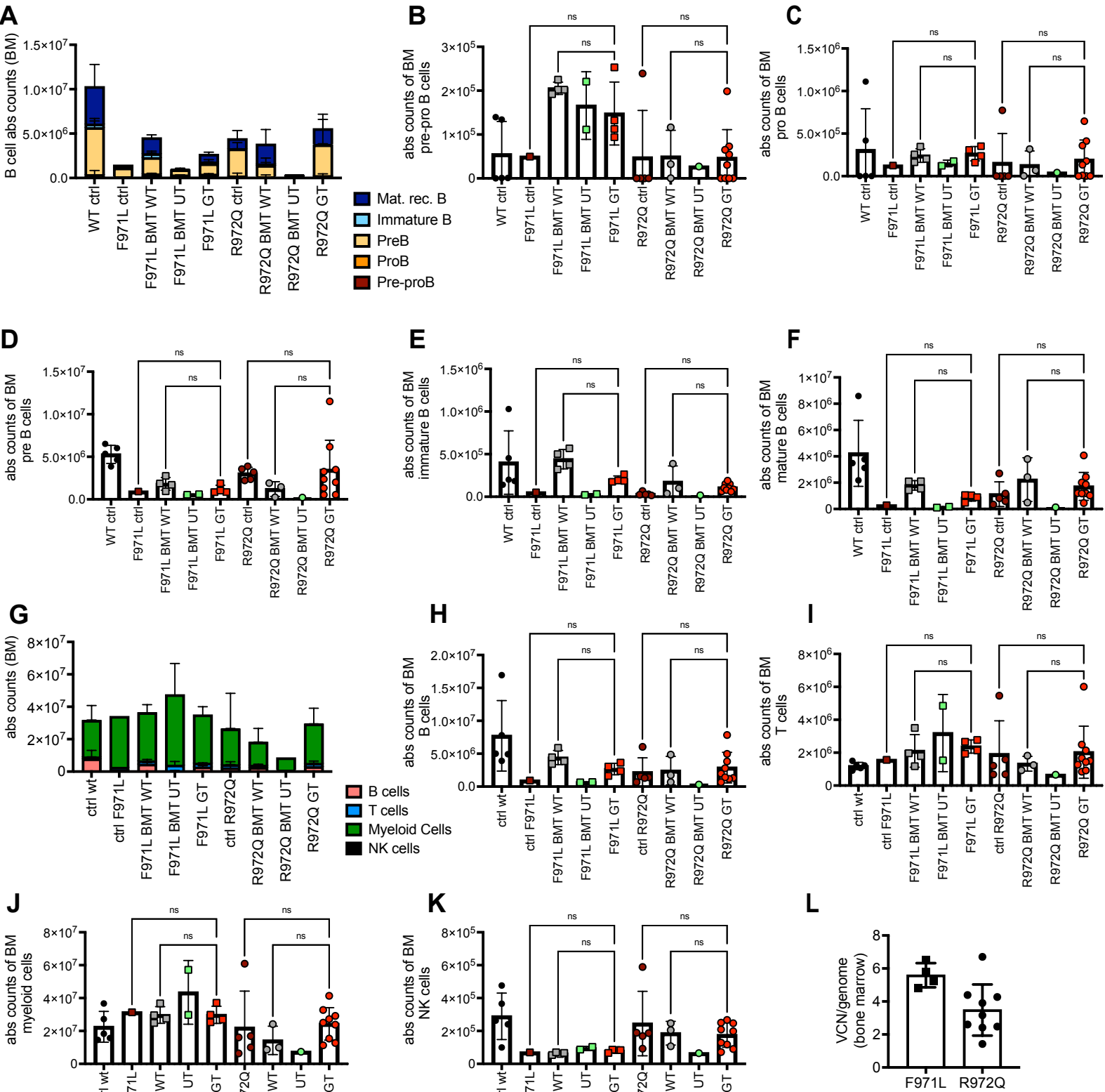

Supplement: Supplementary Figure 8 — Immune cell reconstitution in bone marrow at termination 12 months post-transplant. (A-F). Absolute counts of B cell subsets (A) in bone marrow (BM) of treated and control (ctrl) mice: pre-pro B (B), proB (C), preB (D), immature (E) and mature B cells (F). G-K. Absolute counts of immune subsets (G) in the BM: B (H), T (I), myeloid (J) and natural killer (NK) (K) cells. L. Vector copy number (VCN)/genome in single cell suspensions from bone marrow of hypomorphic gene therapy (GT) mice. WT ctrl, wild-type control (n=5); F971L ctrl, untreated Rag1F971L/F971L (n=1); R972Q ctrl, untreated Rag1R972Q/R972Q (n=5); F971L BMT WT, Rag1F971L/F971L transplanted with WT cells (n=4); R972Q BMT WT, Rag1R972Q/R972Q transplanted with WT cells (n=3); F971L BMT UT, Rag1F971L/F971L transplanted with untransduced cells (n=2); R972Q BMT UT, Rag1R972Q/R972Q transplanted with untransduced cells (n=1); F971L GT, Rag1F971L/F971L transplanted with gene therapy cells (n=3-4); R972Q GT, Rag1R972Q/R972Q transplanted with gene therapy cells (n=8-9). Graphs show mean ± standard deviation (SD). n=2 independent experiments. Statistical analysis (panels (B-F, H-K): non parametric one-way ANOVA: ns not significant p>0.05. [file Image_8.pdf]

# Supplementary figure S9

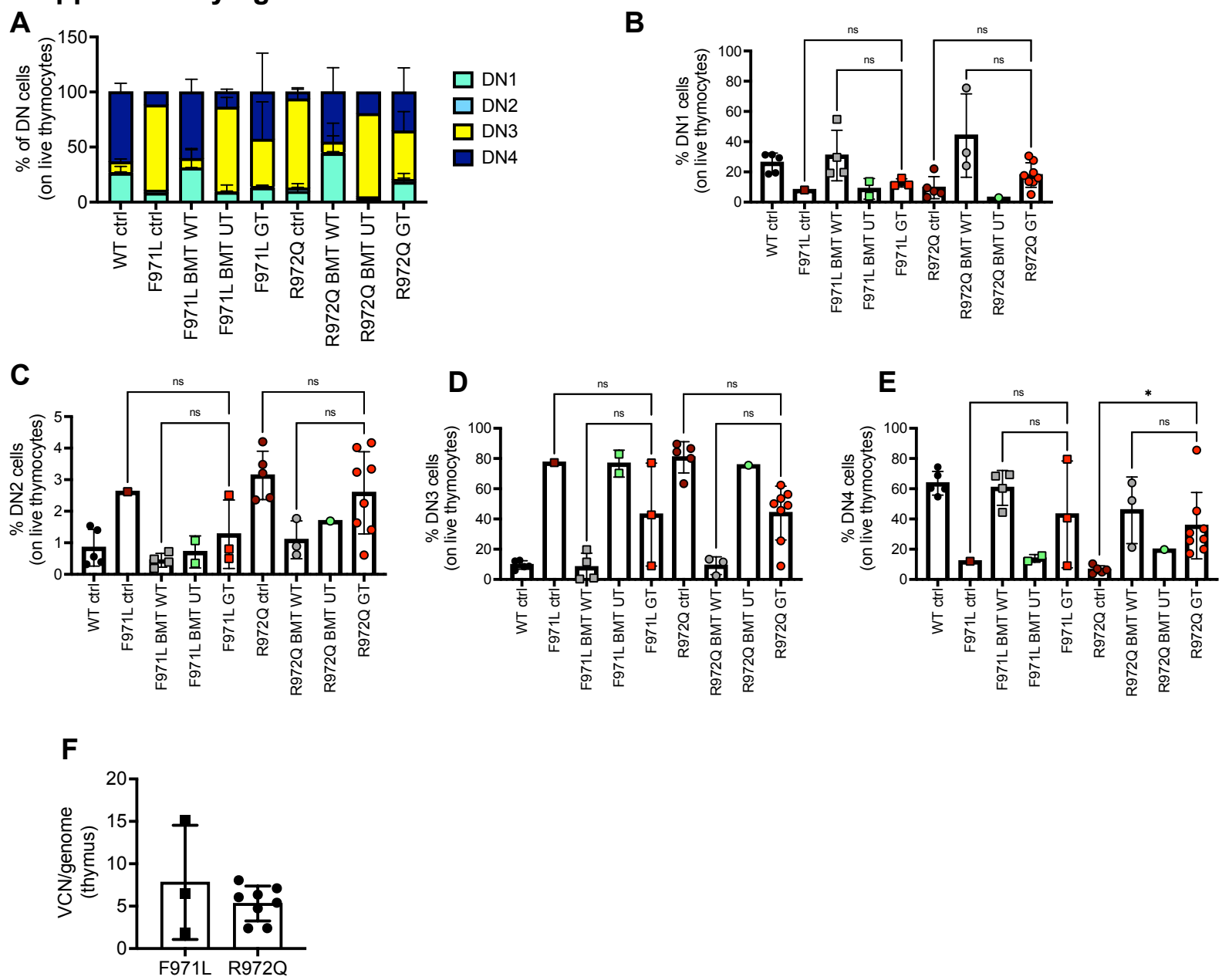

Supplement: Supplementary Figure 9 — Immune cell reconstitution in thymus at termination 12 months post-transplant. (A-E). Frequency of double negative (DN) stages (A) of thymocytes: DN1 (B), DN2 (C), DN3 (D), DN4 (E). (F). Vector copy number (VCN)/genome in single cell suspensions thymus of hypomorphic gene therapy (GT) mice. WT ctrl, wild-type control (n=5); F971L ctrl, untreated Rag1F971L/F971L (n=1); R972Q ctrl, untreated Rag1R972Q/R972Q (n=5); F971L BMT WT, Rag1F971L/F971L transplanted with WT cells (n=4); R972Q BMT WT, Rag1R972Q/R972Q transplanted with WT cells (n=3); F971L BMT UT, Rag1F971L/F971L transplanted with untransduced cells (n=2); R972Q BMT UT, Rag1R972Q/R972Q transplanted with untransduced cells (n=1); F971L GT, Rag1F971L/F971L transplanted with gene therapy cells (n=3-4); R972Q GT, Rag1R972Q/R972Q transplanted with gene therapy cells (n=8-9). Graphs show mean ± standard deviation (SD). n=2. Statistical analysis (panels B-E): non parametric one-way ANOVA: * p<0.05, ns not significant p>0.05. [file Image_9.pdf]

# Supplementary figure S10

**A**

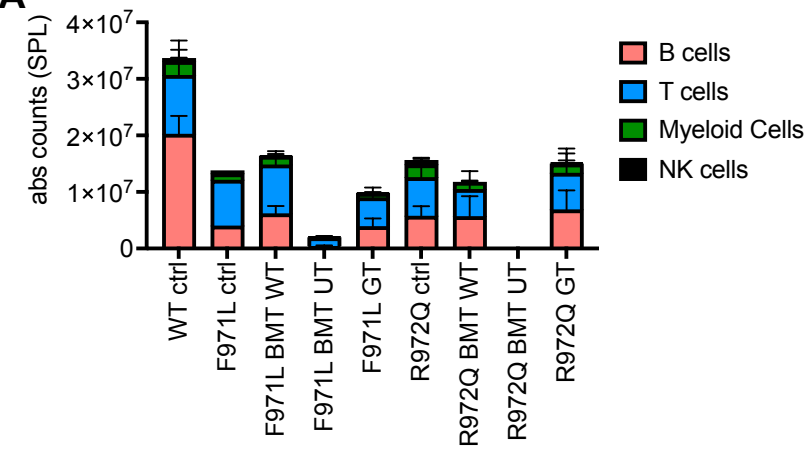

**B**

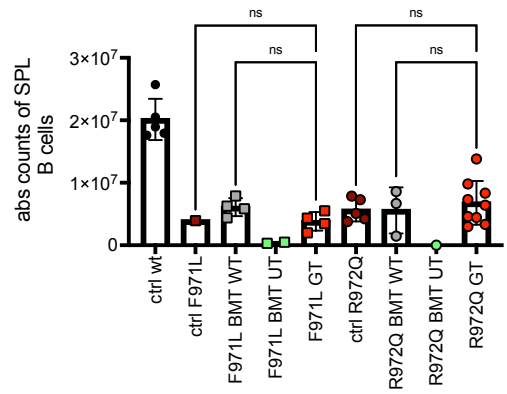

**C**

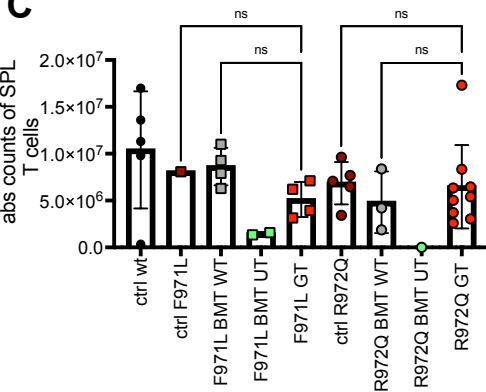

**D**

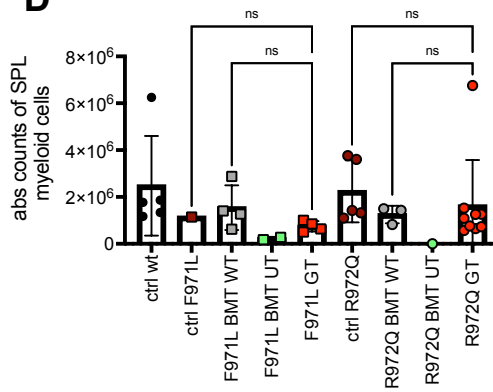

**E**

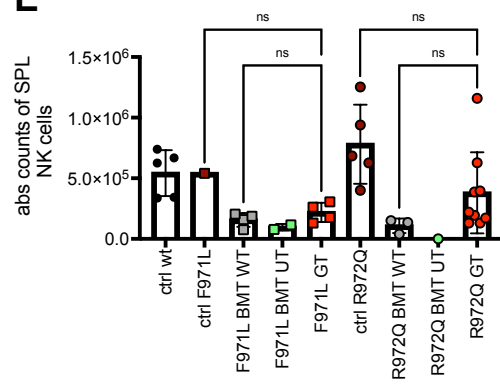

**F**

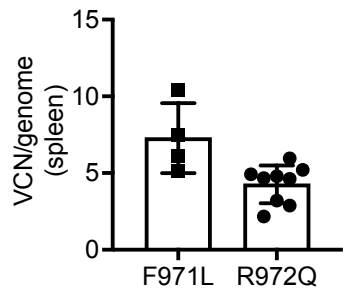

**G**

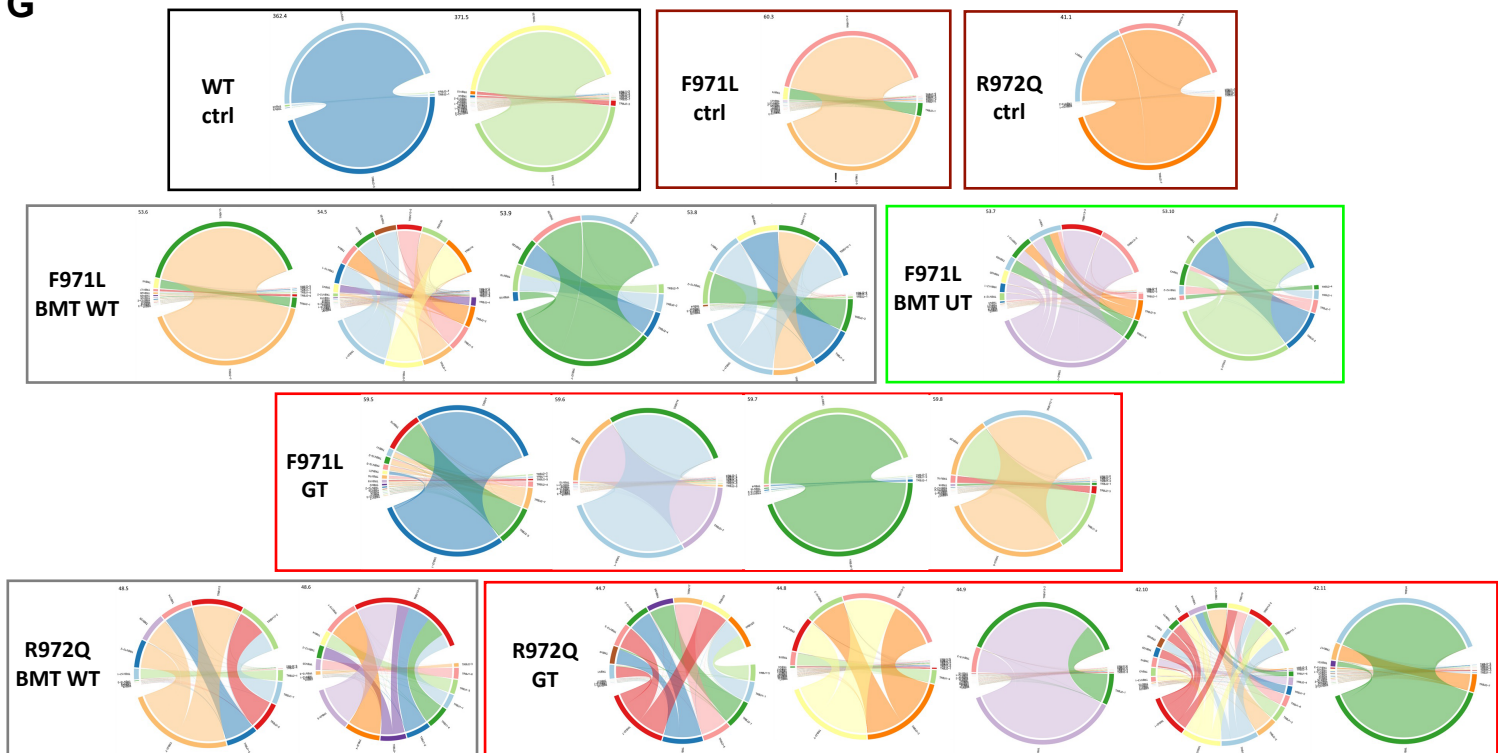

Supplement: Supplementary Figure 10 — Immune cell reconstitution in spleen at termination 12 months post-transplant. (A-E). Absolute counts of immune subsets (A) in the spleen: B (B), T (C), myeloid (D) and natural killer (NK) (E) cells in the spleen. (F). Vector copy number (VCN)/genome in single cell suspensions from spleen of hypomorphic gene therapy (GT) mice. (G). V- and J-gene recombination of T cell receptors (TCR) in splenocytes. Each bow between V- and J-gene represents a clonotype. The broader a bow, the higher relative frequency was detected for the respective clonotype. WT ctrl, wild-type control (n=5); F971L ctrl, untreated Rag1F971L/F971L (n=1); R972Q ctrl, untreated Rag1R972Q/R972Q (n=5); F971L BMT WT, Rag1F971L/F971L transplanted with WT cells (n=4); R972Q BMT WT, Rag1R972Q/R972Q transplanted with WT cells (n=3); F971L BMT UT, Rag1F971L/F971L transplanted with untransduced cells (n=2); R972Q BMT UT, Rag1R972Q/R972Q transplanted with untransduced cells (n=1); F971L GT, Rag1F971L/F971L transplanted with gene therapy cells (n=3-4); R972Q GT, Rag1R972Q/R972Q transplanted with gene therapy cells (n=8-9). Graphs show mean ± standard deviation (SD). n=2 (panels A-F) or n=1 (panel G) independent experiments. Statistical analysis (panels B-E): non parametric one-way ANOVA: * p<0.05, ns not significant p>0.05. [file Image_10.pdf]

# Supplementary figure S11

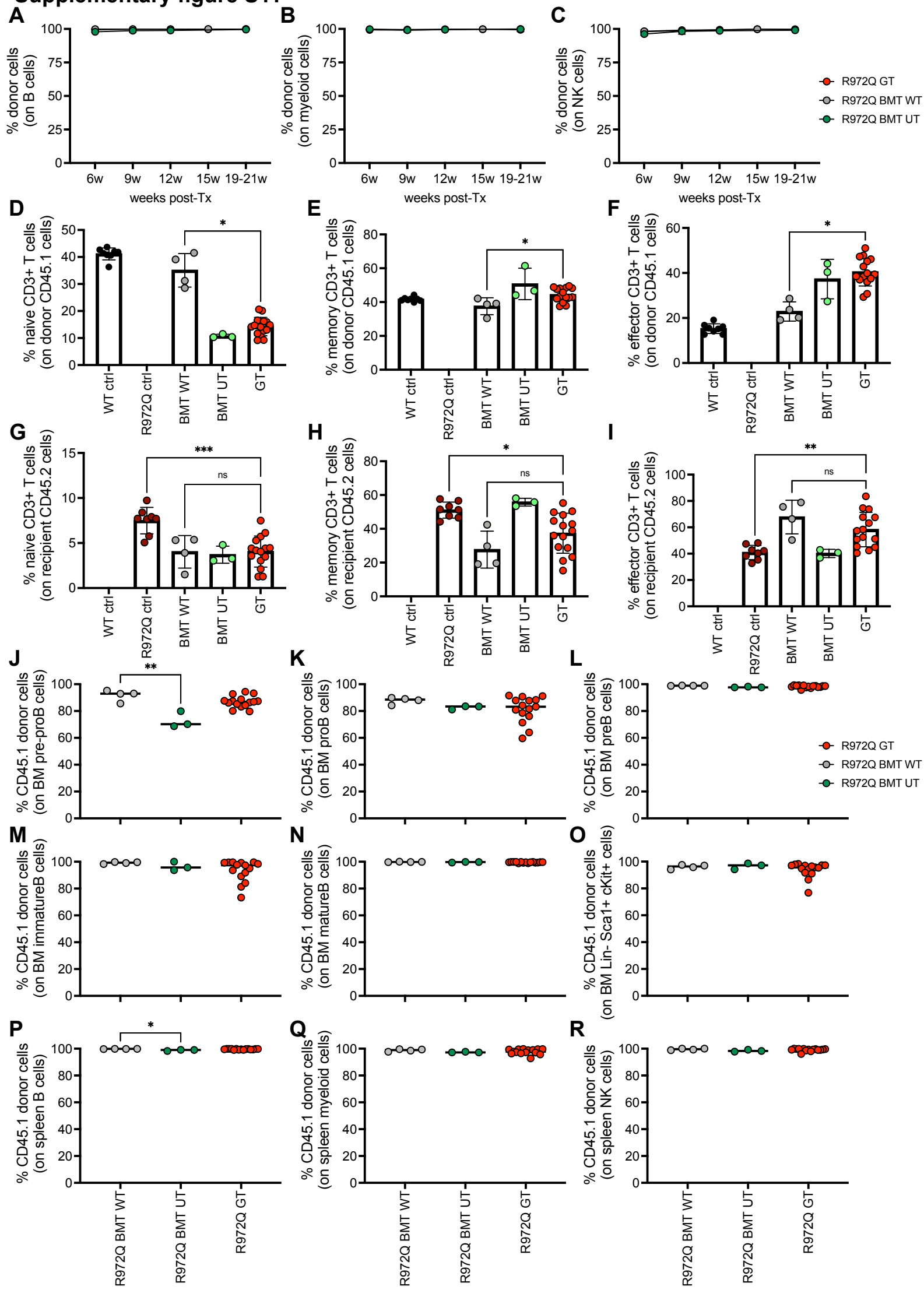

Supplement: Supplementary Figure 11 — Donor chimerism in CD45-mismatched transplants, terminated 6 months post-transplant. (A-C). Chimerism of donor cells (CD45.1) over time in the B (A), myeloid (B) and NK (C) cells of peripheral blood. (D-F). Frequency of T cells subsets in donor CD45.1 cells of the spleen: naïve (D), effector (E) and memory (F) T cells. G-I. Frequency of T cells subsets in recipient CD45.2 cells of the spleen: naïve (G), effector (H) and memory (I) T cells. J-N. Chimerism of CD45.1+ donor cells in the pre-proB (J), proB (K), preB (L), immature (M) and mature recirculating B (N) cells of the bone marrow. (O). Chimerism of CD45.1+ donor cells in the Lin- Sca1+ cKit+ progenitor cells of the bone marrow. (P-R). Chimerism of donor cells (CD45.1) in the B (P), myeloid (Q) and NK (R) cells of the spleen. R972Q BMT WT, Rag1R972Q/R972Q transplanted with WT cells (n=4); R972Q BMT UT, Rag1R972Q/R972Q transplanted with untransduced cells (n=3); R972Q GT, Rag1R972Q/R972Q transplanted with gene therapy cells (n=15). n=2 independent experiments. Graphs show mean ± SD (panels A-I) or median (panels J-R). Statistical analysis: non parametric one-way ANOVA, * p<0.05, ** p<0.01, *** p<0.001, ns not significant p>0.05. In panels J-R, only statistically significant comparisons are shown. [file Image_11.pdf]

# Supplementary figure S12

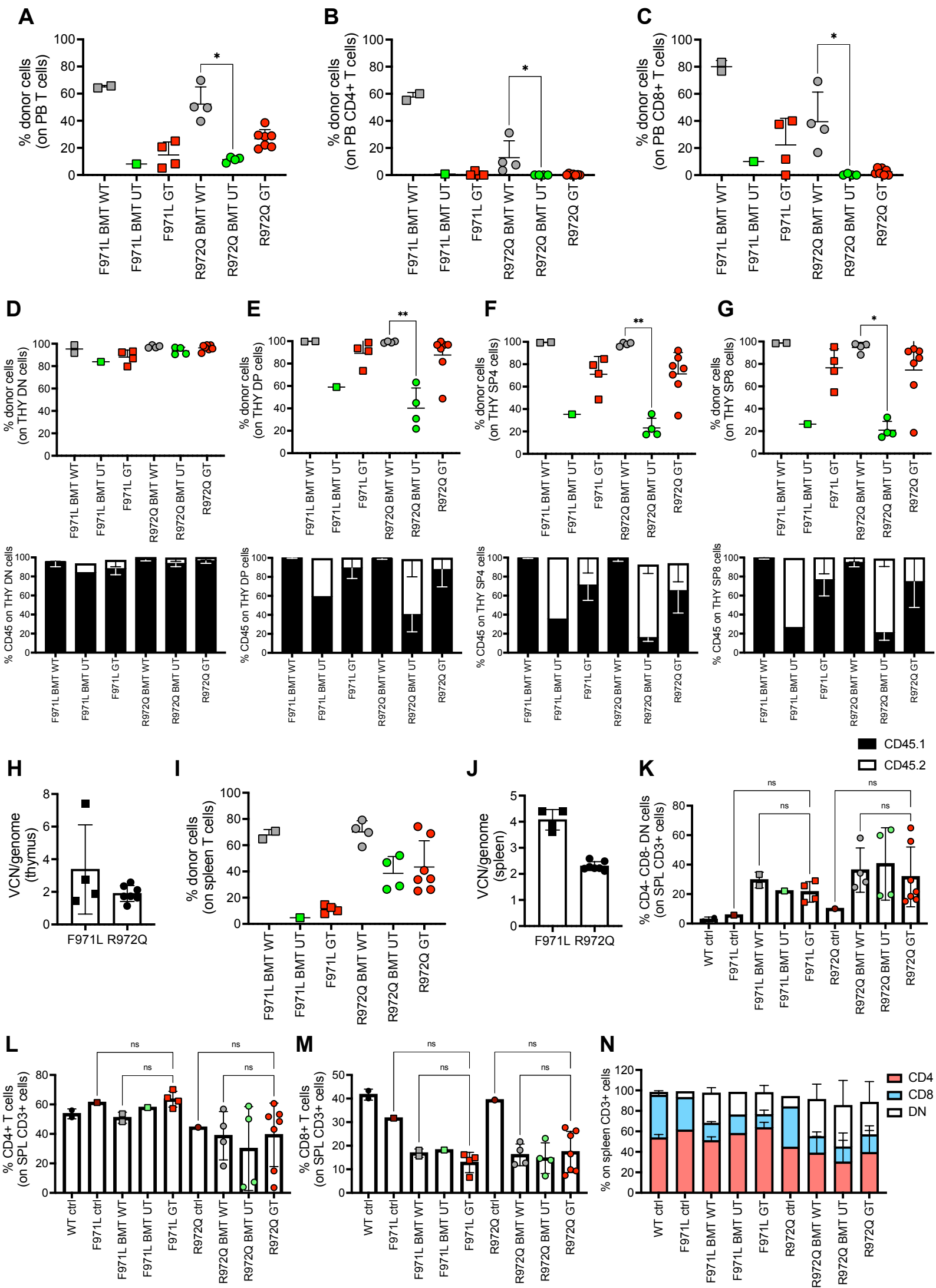

Supplement: Supplementary Figure 12 — Short-term donor chimerism in CD45-mismatched transplants, terminated 3 weeks post-transplant. (A-C). Chimerism of donor cells (CD45.1) in the total T (A), CD4+ T (B) and CD8+ T (C) cells of peripheral blood. (D-G). Chimerism of donor cells in the double negative (DN, (D), double positive (DP, (E), single positive 4 (SP4, (F) and single positive 8 (SP8, (G) subsets of the thymus. Graphs show frequency of donor CD45.1 cells (top) or fraction of donor CD45.1+ and recipient CD45.2+ cells (bottom). (H). Vector copy number (VCN)/genome in single cell suspensions from the thymus of hypomorphic gene therapy (GT) mice. (I). Frequency of donor CD45.1 cells in the T cells of the spleen. (J). Vector copy number (VCN)/genome in single cell suspensions from spleen of hypomorphic gene therapy (GT) mice. (K-N). Distribution of CD4- CD8- (K), CD4+ (L), CD8+ (M) T cells (N) of the spleen. WT ctrl, wild-type control (n=2); F971L ctrl, untreated Rag1F971L/F971L (n=1); R972Q ctrl, untreated Rag1R972Q/R972Q (n=1); F971L BMT WT, Rag1F971L/F971L transplanted with WT cells (n=2); R972Q BMT WT, Rag1R972Q/R972Q transplanted with WT cells (n=4); F971L BMT UT, Rag1F971L/F971L transplanted with untransduced cells (n=1); R972Q BMT UT, Rag1R972Q/R972Q transplanted with untransduced cells (n=4); F971L GT, Rag1F971L/F971L transplanted with gene therapy cells (n=4); R972Q GT, Rag1R972Q/R972Q transplanted with gene therapy cells (n=7). Graphs show mean ± standard deviation (SD). n=2 independent experiments. Statistical analysis: non parametric one-way ANOVA, * p<0.05, ** p<0.01, ns not significant p>0.05. In panels A-G and I, only statistically significant comparisons are shown; in panels K-M, all performed comparisons are shown. [file Image_12.pdf]
