# Supplementary material for: Partial correction of immunodeficiency by lentiviral vector gene therapy in mouse models carrying Rag1 hypomorphic mutations
Source: Front Immunol. 2023 Nov 13;14:1268620. doi: 10.3389/fimmu.2023.1268620 (PMC10679457; doi:10.3389/fimmu.2023.1268620)
Supplement: Supplementary Figure 13 — Integration site analysis. (A, B). Cumulative retrieval frequencies of the ten most prominent integration sites (ISs) detected in the thymus and spleen of GT animals with abnormal (A) or normal (B) thymic tissue. Vector copy numbers/genome (VCNs) of thymus (Thy) and spleen (Spl) are shown. Sequence data from all S-EPTS/LM-PCR (shearing extension primer tag selection ligation-mediated PCR) amplicons are combined. Sequence count of the ten most prominent ISs (colour) and sequence count of all remaining ISs (grey) as well as total sequence count from all amplicons are shown at the bottom of the table. RefSeq names of genes located closest to the respective IS are given in the table. Relative sequence count contributions of the ten most prominent ISs and all remaining mappable IS are shown (frequency). [file Image_13.pdf]

Supplementary figure S13

A

Thymus / Spleen

39.1 F971L GT
VCN Thy 1.9
VCN Spl 0.2

Thymus

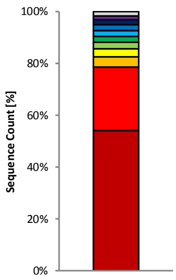

Rank	Gene Name	Frequency [%]	Location
Top1	Sik3	53.912	9+46051231
Top2	Brd4	24.561	17-32286543
Top3	Acvr2a	3.909	2-48382212
Top4	Ptpre	3.129	7-135561758
Top5	Runx2	2.521	17+44578170
Top6	Hpse2	2.334	19+43297866
Top7	Magi2	2.329	5+20487422
Top8	Ankrd32	2.235	13+77112838
Top9	Bahcc1	2.081	11-120289117
Top10	Lcorl	1.158	5-45743976
#All Other mapp. IS	49	1.831	
Seq Count 10 Strongest	212901		
Seq Count all other mapp. IS	3971		
Total Seq Counts Used	216872		

Spleen

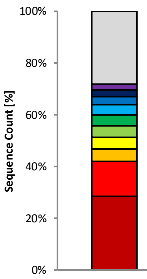

Rank	Gene Name	Frequency [%]	Location
Top1	Sik3	28.26	9+46051231
Top2	Brd4	13.524	17-32286543
Top3	4933402J10Rik	4.826	5+59984526
Top4	Ptpre	4.621	7-135561758
Top5	Runx2	4.51	17+44578170
Top6	Bahcc1	4.154	11-120289117
Top7	Rsrc2	4.03	5+123739437
Top8	Tti2	3.112	8-31160288
Top9	Larp4b	2.63	13-9100118
Top10	Shisa5	2.134	9+109054279
#All Other mapp. IS	506	28.199	
Seq Count 10 Strongest	186373		
Seq Count all other mapp. IS	73196		
Total Seq Counts Used	259569		

39.3 F971L GT
VCN Thy 0.1
VCN Spl 3.1

Thymus

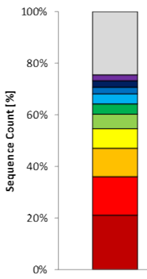

Rank	Gene Name	Frequency [%]	Location
Top1	Arpp21	20.935	9+112205686
Top2	Maml1	15.153	11-50283675
Top3	Zdhhc9	11.01	X+48167366
Top4	Rspry1	7.511	8+94623980
Top5	Kdm4b	5.554	17+56339937
Top6	AU022793	4.087	15+40002755
Top7	Atad2b	3.785	12-4941353
Top8	Actn1	2.744	12+80228537
Top9	Ski	2.403	4+155225423
Top10	Rsbn1l	2.148	5-20893788
#All Other mapp. IS	589	24.67	
Seq Count 10 Strongest	92593		
Seq Count all other mapp. IS	30323		
Total Seq Counts Used	122916		

Spleen

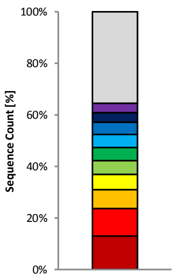

Rank	Gene Name	Frequency [%]	Location
Top1	Zdhhc9	12.878	X+48167366
Top2	Arpp21	10.804	9+112205686
Top3	Atad2b	7.254	12-4941353
Top4	Rspry1	5.831	8+94623980
Top5	Ski	5.373	4+155225423
Top6	Actn1	5.153	12+80228537
Top7	Maml1	5.135	11-50283675
Top8	Kdm4b	4.851	17+56339937
Top9	Lipi	3.636	16-75358560
Top10	Pcm1	3.464	8-41319232
#All Other mapp. IS	239	35.621	
Seq Count 10 Strongest	73817		
Seq Count all other mapp. IS	40843		
Total Seq Counts Used	114660		

24.5 R972Q GT
VCN Thy 0.3
VCN Spl 3.6

Thymus

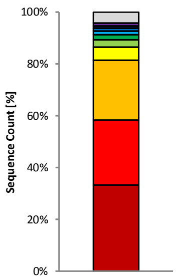

Rank	Gene Name	Frequency [%]	Location
Top1	Ofd1	33.136	X+166392303
Top2	Mir195b	25.027	2-56329925
Top3	Samm50	23.222	15+84217526
Top4	Tpr	5.195	1-150439247
Top5	Sgms1	2.742	19+32253892
Top6	Brd4	2.069	17+32278136
Top7	Syne1	1.158	10-5529901
Top8	Dnajc10	1.134	2+80332037
Top9	Ccnd3	1.066	17+47513854
Top10	Pde4d	0.94	13+109434826
#All Other mapp. IS	88	4.311	
Seq Count 10 Strongest	69052		
Seq Count all other mapp. IS	3111		
Total Seq Counts Used	72163		

Spleen

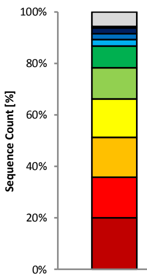

Rank	Gene Name	Frequency [%]	Location
Top1	Ofd1	19.89	X+166392303
Top2	Mir195b	15.806	2-56329925
Top3	Abca13	15.441	11+9538716
Top4	Samm50	14.992	15+84217526
Top5	Itch	12.027	2+155168675
Top6	Usp54	8.524	14+20633176
Top7	Tpr	2.626	1-150439247
Top8	Sgms1	2.287	19+32253892
Top9	Ptpra	2.125	2+130511261
Top10	Brd4	0.74	17+32278136
#All Other mapp. IS	156	5.542	
Seq Count 10 Strongest	217167		
Seq Count all other mapp. IS	12742		
Total Seq Counts Used	229909		

59.5 F971L GT
VCN Thy 13.4
VCN Spl 10.4

Thymus

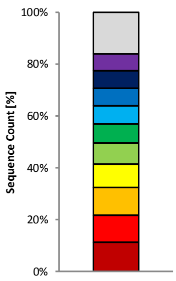

Rank	Gene Name	Frequency [%]	Location
Top1	Atad2	11.069	15-58120355
Top2	Stk24	10.581	14-121361835
Top3	Nfix	10.568	8-84764560
Top4	4933402C06Rik	9.022	7-40150779
Top5	Impg1	8.243	9+80380981
Top6	Ccdc85a	7.324	11-28374460
Top7	Hnrnpk	7.074	13-58390922
Top8	Dock11	6.844	X-36024085
Top9	Zbtb46	6.716	2-181417833
Top10	Ppp3ca	6.491	3-136767995
#All Other mapp. IS	69	16.068	
Seq Count 10 Strongest	96040		
Seq Count all other mapp. IS	18386		
Total Seq Counts Used	114426		

Spleen

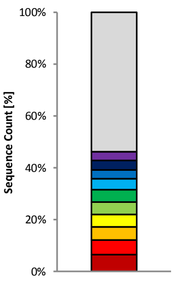

Rank	Gene Name	Frequency [%]	Location
Top1	Impg1	6.424	9+80380981
Top2	Suclg2	5.514	6-95596663
Top3	Dock11	5.065	X-36024085
Top4	4933402C06Rik	5.018	7-40150779
Top5	Skint2	4.798	4+112561004
Top6	Nfix	4.591	8-84764560
Top7	Stk24	4.183	14-121361835
Top8	Hnrnpk	3.639	13-58390922
Top9	Ppp3ca	3.495	3-136767995
Top10	Atad2	3.471	15-58120355
#All Other mapp. IS	200	53.802	
Seq Count 10 Strongest	57038		
Seq Count all other mapp. IS	66426		
Total Seq Counts Used	123464		

79.16 R972Q GT
VCN Thy 0.1
VCN Spl 2.2

Thymus

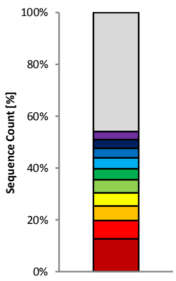

Rank	Gene Name	Frequency [%]	Location
Top1	Chd1	12.543	17-15732665
Top2	Deptor	6.99	15+55173601
Top3	Dsc3	5.785	18+19742232
Top4	Zfr	5.164	15-12173699
Top5	Tank	4.839	2-61602270
Top6	Ccdc59	4.42	10-105957954
Top7	Emr4	4.062	17+55737354
Top8	Frrs1	3.712	3+116894688
Top9	Atr	3.413	9+95917199
Top10	9330175E14Rik	2.979	8+94438089
#All Other mapp. IS	115	46.093	
Seq Count 10 Strongest	19271		
Seq Count all other mapp. IS	16478		
Total Seq Counts Used	35749		

Spleen

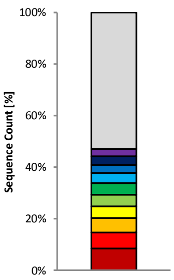

Rank	Gene Name	Frequency [%]	Location
Top1	Kdm6a	8.371	X-18188347
Top2	Spcs3	6.058	8-54524898
Top3	Deptor	5.867	15+55173601
Top4	Tank	4.487	2-61602270
Top5	Pan3	4.473	5-147492097
Top6	Tmem243	4.406	5-9104799
Top7	Cdc25a	3.99	9+109878402
Top8	Zfr	3.256	15-12173699
Top9	Hmgxb4	3.147	8-75012436
Top10	Mmp16	2.953	4-17885556
#All Other mapp. IS	100	52.992	
Seq Count 10 Strongest	43921		
Seq Count all other mapp. IS	49512		
Total Seq Counts Used	93433		

127.1 F971L GT
VCN Thy 0.04
VCN Spl 3.2

Thymus

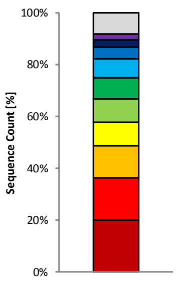

Rank	Gene Name	Frequency [%]	Location
Top1	Baiap2	19.803	11+119985425
Top2	Gga3	16.446	11-115602102
Top3	Gls	12.34	1-52227189
Top4	Nbas	9.114	12-13289632
Top5	Tex2	8.922	11-106555212
Top6	Nipbl	8.209	15+8405455
Top7	Smc5	7.402	19+23235599
Top8	Clint1	4.493	11-45864505
Top9	Prkacb	2.732	3+146752189
Top10	Asxl1	2.422	2+153367732
#All Other mapp. IS	120	8.117	
Seq Count 10 Strongest	54087		
Seq Count all other mapp. IS	4778		
Total Seq Counts Used	58865		

Spleen

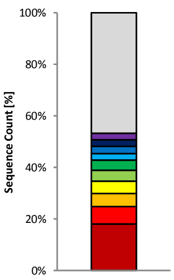

Rank	Gene Name	Frequency [%]	Location
Top1	Rabgap1l	17.899	1-160289581
Top2	Rbx1	6.887	15-81481756
Top3	4930559C10Rik	4.924	12+93179365
Top4	Micu3	4.82	8-40344154
Top5	Klhl1	4.379	14-96017897
Top6	Nipsnap3b	3.721	4+53018818
Top7	Baz2b	2.714	2+60081980
Top8	Tdrd3	2.641	14+87480416
Top9	Prl4a1	2.589	13+28046407
Top10	Acsl1	2.549	8+46509134
#All Other mapp. IS	155	46.877	
Seq Count 10 Strongest	74350		
Seq Count all other mapp. IS	65609		
Total Seq Counts Used	139959		

127.2 F971L GT
VCN Thy 0.01
VCN Spl 2.2

Thymus

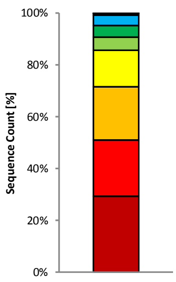

Rank	Gene Name	Frequency [%]	Location
Top1	Agmo	29.195	12+37248692
Top2	Arhgap35	21.764	7-16586014
Top3	Xrcc5	20.537	1-72336184
Top4	Triqk	14.219	4+12927277
Top5	Dcc	4.984	18-72325194
Top6	Qk	4.439	17+10230375
Top7	Lacc1	4.13	14+76879280
Top8	Kpna3	0.577	14-61434382
Top9	4921531P14Rik	0.014	18+83488039
Top10	Notch1	0.006	2+26458599
#All Other mapp. IS	91	0.135	
Seq Count 10 Strongest	86494		
Seq Count all other mapp. IS	117		
Total Seq Counts Used	86611		

Spleen

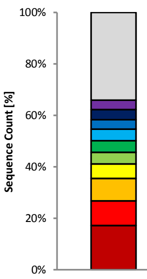

Rank	Gene Name	Frequency [%]	Location
Top1	Arhgap35	17.108	7-16586014
Top2	Xrcc5	9.612	1-72336184
Top3	Wac	8.846	18-78875262
Top4	Asxl3	5.438	18+22460890
Top5	Triqk	4.598	4+12927277
Top6	Ugp2	4.462	11-21347341
Top7	Sec23a	4.408	12+58958827
Top8	Lacc1	3.897	14+76879280
Top9	Qk	3.893	17-10235448
Top10	Myo5a	3.537	9+75161359
#All Other mapp. IS	106	34.201	
Seq Count 10 Strongest	85466		
Seq Count all other mapp. IS	44423		
Total Seq Counts Used	129889		

B

Thymus / Spleen

39.9 F971L GT
VCN Thy 1.9
VCN Spl 2.9

Thymus

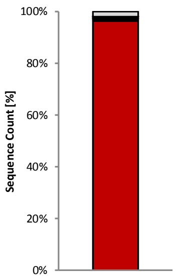

Rank	Gene Name	Frequency [%]	Location
Top1	Nfam1	96.456	15-83021697
Top2	Ptprk	0.42	10-28480627
Top3	Gmps	0.21	3+64039303
Top4	Pten	0.209	19-32786482
Top5	Cd320	0.168	17-33848792
Top6	Ctsq	0.159	13-61022933
Top7	Mef2c	0.154	13+83528863
Top8	Schip1	0.138	3+68158072
Top9	Pik3r1	0.131	13-101699862
Top10	Diap3	0.12	14+86995269
#All Other mapp. IS	99	1.835	

Seq Count 10 Strongest	73407
Seq Count all other mapp. IS	1372
Total Seq Counts Used	74779

Spleen

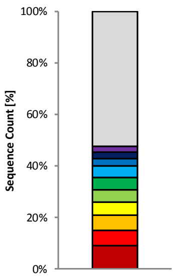

Rank	Gene Name	Frequency [%]	Location
Top1	Bicd2	9	13+49359328
Top2	Pten	5.906	19-32786482
Top3	Ctsq	5.727	13-61022933
Top4	Mef2c	5.283	13+83528863
Top5	Ptprk	4.73	10-28480627
Top6	E230016M11Rik	4.705	6-67055964
Top7	A630089N07Rik	4.5	16+98021198
Top8	Diap3	2.784	14+86995269
Top9	Pik3r1	2.702	13-101699862
Top10	Vmp1	2.289	11-86625245
#All Other mapp. IS	202	52.374	

Seq Count 10 Strongest	47861
Seq Count all other mapp. IS	52633
Total Seq Counts Used	100494

128.5 F971L GT
VCN Thy 5.7
VCN Spl 3.5

Thymus

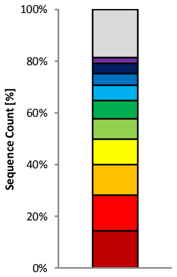

Rank	Gene Name	Frequency [%]	Location
Top1	1700016G22Rik	14.306	13+5739501
Top2	Cst3	13.668	2-148870723
Top3	Scaf4	11.955	16-90260797
Top4	Taf4b	10.012	18-14813582
Top5	Stim2	7.708	5+54173522
Top6	Suclg2	7.157	6-95611931
Top7	Csnk2a2	5.792	8-95466744
Top8	Scaf4	4.504	16+90260797
Top9	Ghitm	3.906	14-37328259
Top10	Hipk3	2.503	2-104452947
#All Other mapp. IS	41	18.489	

Seq Count 10 Strongest	36831
Seq Count all other mapp. IS	8354
Total Seq Counts Used	45185

Spleen

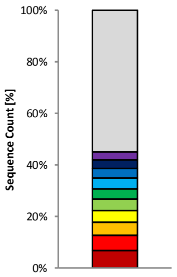

Rank	Gene Name	Frequency [%]	Location
Top1	Taf4b	6.721	18-14813582
Top2	Scaf4	5.971	16-90260802
Top3	Efna5	4.974	17-62819607
Top4	1700016G22Rik	4.618	13+5739501
Top5	Arhgap32	4.352	9+32201513
Top6	Cst3	4.114	2-148870723
Top7	Csnk2a2	4.046	8-95466744
Top8	Itga8	3.606	2-12224769
Top9	Serbp1	3.43	6-67287700
Top10	Arnt2	3.309	7-84390680
#All Other mapp. IS	184	54.859	

Seq Count 10 Strongest	94881
Seq Count all other mapp. IS	115308
Total Seq Counts Used	210189

39.2 F971L GT
VCN Thy 0.01
VCN Spl 1.9

Thymus

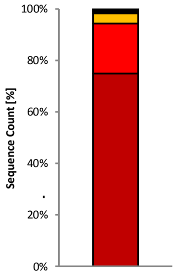

Rank	Gene Name	Frequency [%]	Location
Top1	Tomm70a	74.795	16-57134550
Top2	Lyst	19.696	13-13593944
Top3	Nova1	3.846	12-46763826
Top4	Csrp2bp	0.525	2-144371039
Top5	Flrt2	0.428	12+95673759
Top6	Fndc3a	0.221	14-72692535
Top7	4930470O06Rik	0.209	8+60157584
Top8	Tab2	0.149	10-7914344
Top9	Nabp1	0.095	1-51474427
Top10	Nmt2	0.009	2+3314616
#All Other mapp. IS	10	0.027	

Seq Count 10 Strongest	109966
Seq Count all other mapp. IS	30
Total Seq Counts Used	109996

Spleen

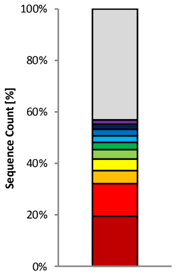

Rank	Gene Name	Frequency [%]	Location
Top1	Svil	19.447	18-5097312
Top2	Tomm70a	12.502	16-57134550
Top3	Cyp2j9	5.069	4+96573736
Top4	4930470O06Rik	4.489	8+60157584
Top5	Flrt2	3.78	12+95673759
Top6	Ube2f	2.884	1+91276138
Top7	Fndc3a	2.574	14-72692535
Top8	Stap1	2.572	5-86100894
Top9	Nova1	1.801	12-46763826
Top10	Mark3	1.745	12+111632410
#All Other mapp. IS	283	43.137	

Seq Count 10 Strongest	65327
Seq Count all other mapp. IS	49558
Total Seq Counts Used	114885

25.2 R972Q GT
VCN Thy 1.1
VCN Spl 2.9

Thymus

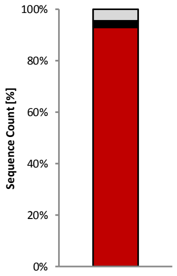

Rank	Gene Name	Frequency [%]	Location
Top1	Xrcc6	92.881	15+82019722
Top2	Inpp5f	0.399	7+128599845
Top3	Ndufa8	0.335	2+36043235
Top4	Rps29	0.33	12+69130448
Top5	Snx29	0.304	16+11458715
Top6	Gm20139	0.293	10+19232835
Top7	Arl4a	0.292	12-40029388
Top8	Trove2	0.236	1-143751144
Top9	Kif16b	0.222	2+142680586
Top10	Nsun3	0.204	16-62500503
#All Other mapp. IS	139	4.504	

Seq Count 10 Strongest	199379
Seq Count all other mapp. IS	9404
Total Seq Counts Used	208783

Spleen

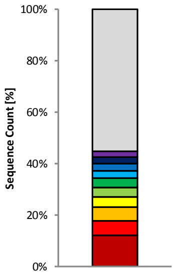

Rank	Gene Name	Frequency [%]	Location
Top1	Xrcc6	12.149	15+82019722
Top2	Ccdc144b	5.481	3-36024168
Top3	Kif16b	5.35	2+142680586
Top4	Inpp5f	3.872	7+128599845
Top5	Scaf8	3.831	17+3149470
Top6	Prdm16	3.692	4-154529722
Top7	Arl4a	2.787	12-40029388
Top8	Ctif	2.648	18+75475894
Top9	Trove2	2.536	1-143751144
Top10	Thsd7a	2.476	6+12498749
#All Other mapp. IS	224	55.178	

Seq Count 10 Strongest	58256
Seq Count all other mapp. IS	71715
Total Seq Counts Used	129971

59.6 F971L
VCN Thy 15.1
VCN Spl 7.5

Thymus

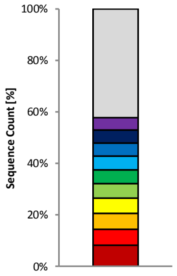

Rank	Gene Name	Frequency [%]	Location
Top1	Fam198b	8.039	3+80055501
Top2	Prpf39	6.327	12+65054525
Top3	Golim4	6.091	3+75992539
Top4	Lamp2	5.855	X-38435082
Top5	Nova1	5.606	12+46810721
Top6	Pid1	5.402	1-84235621
Top7	Rnf180	5.381	13-105252564
Top8	Gm6634	5.204	3-70804939
Top9	Nr3c1	5.108	18-39461435
Top10	Phlpp1	4.736	1+106190116
#All Other mapp. IS	71	42.251	

Seq Count 10 Strongest	66200
Seq Count all other mapp. IS	48434
Total Seq Counts Used	114634

Spleen

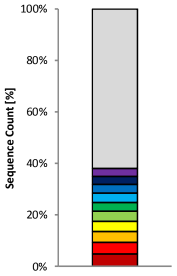

Rank	Gene Name	Frequency [%]	Location
Top1	Cenpc1	4.818	5+86026262
Top2	Prpf39	4.39	12+65054525
Top3	Fam198b	4.26	3+80055501
Top4	Phlpp1	4.035	1+106190116
Top5	Nr3c1	3.753	18-39461435
Top6	Pid1	3.517	1-84235621
Top7	Cr1l	3.48	1+195117924
Top8	Utrn	3.421	10-12755131
Top9	4930440C22Rik	3.113	1+94787623
Top10	Rbm17	3.106	2-11604906
#All Other mapp. IS	300	62.107	

Seq Count 10 Strongest	62393
Seq Count all other mapp. IS	102263
Total Seq Counts Used	164656

44.8 R972Q GT
VCN Thy 5.4
VCN Spl 4.6

Thymus

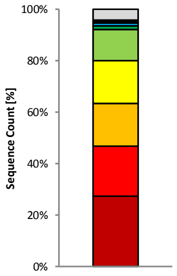

Rank	Gene Name	Frequency [%]	Location
Top1	Dgkd	27.231	1-87858469
Top2	Slc33a1	19.476	3-63958909
Top3	Samsn1	16.762	16-75958941
Top4	Tcf12	16.603	9+72021018
Top5	Stag1	11.993	9-100704540
Top6	Uhrf1bp1l	1.527	10+89811648
Top7	Mink1	1.082	11-70588923
Top8	Trps1	0.412	15+51024130
Top9	Cdk7	0.362	13+100717789
Top10	4930513D17Rik	0.357	5-39573455
#All Other mapp. IS	58	4.195	

Seq Count 10 Strongest	156839
Seq Count all other mapp. IS	6868
Total Seq Counts Used	163707

Spleen

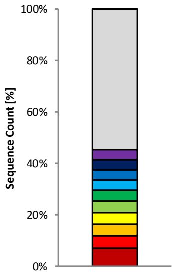

Rank	Gene Name	Frequency [%]	Location
Top1	Rel	7.023	11-23744220
Top2	Uhrf1bp1l	4.635	10+89811648
Top3	Lphn3	4.616	5-81254717
Top4	4930598F16Rik	4.55	1-95740405
Top5	Rb1	4.538	14+73282918
Top6	Hes1	4.198	16-30134559
Top7	Atp10a	3.958	7+58811531
Top8	4930513D17Rik	3.934	5-39573455
Top9	1700016G22Rik	3.875	13+5770123
Top10	Thrb	3.842	14-17912969
#All Other mapp. IS	218	54.831	

Seq Count 10 Strongest	64724
Seq Count all other mapp. IS	78569
Total Seq Counts Used	143293

79.1 R972Q GT
VCN Thy 6.1
VCN Spl 4.8

Thymus

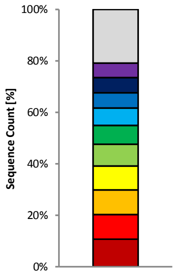

Rank	Gene Name	Frequency [%]	Location
Top1	Dab2ip	10.626	2+35709983
Top2	Macf1	9.571	4+123530319
Top3	Pou2f1	9.52	1+165925102
Top4	Gm597	9.399	1-28057819
Top5	Uggt1	8.383	1+36229087
Top6	Tmpo	7.413	10+91164038
Top7	Dock10	6.727	1-80566641
Top8	Pcm1	5.952	8-41257733
Top9	Fbrs	5.929	7+127483968
Top10	Elavl4	5.656	4+110261798
#All Other mapp. IS	85	20.824	

Seq Count 10 Strongest	79334
Seq Count all other mapp. IS	20865
Total Seq Counts Used	100199

Spleen

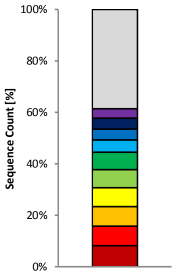

Rank	Gene Name	Frequency [%]	Location
Top1	Gm597	8.114	1-28057819
Top2	Crispld1	7.665	1+17774560
Top3	Macf1	7.458	4+123530319
Top4	Gm6634	7.304	3+70798297
Top5	Erap1	7.25	13+74646362
Top6	Uggt1	6.564	1+36229087
Top7	Pou2f1	4.939	1+165925102
Top8	Ube2e3	4.233	2-79065658
Top9	U2af2	4.148	7+5067534
Top10	Cep192	3.794	18-67858528
#All Other mapp. IS	118	38.531	

Seq Count 10 Strongest	54013
Seq Count all other mapp. IS	33858
Total Seq Counts Used	87871
