# Supplementary material for: Partial correction of immunodeficiency by lentiviral vector gene therapy in mouse models carrying Rag1 hypomorphic mutations
Source: Front Immunol. 2023 Nov 13;14:1268620. doi: 10.3389/fimmu.2023.1268620 (PMC10679457; doi:10.3389/fimmu.2023.1268620)
Supplement: Supplementary file 14 [file Table_1.pdf]

Supplementary Table 1. Integration sites (IS) near Proto-Oncogenes known for severe adverse events in previous trials

Sample	Tissue	IS near Proto-Oncogene	Retrieval frequency [%]
F971L GT 39.1	Spleen	LMO2	0.019
F971L GT 39.3	Thymus	LMO2	0.540
F971L GT 39.3	Spleen	LMO2	0.007
R972Q GT 44.8	Spleen	MECOM	0.119
R972Q GT 44.8	Thymus	MECOM	0.020
R972Q GT 79.16	Thymus	MECOM	0.006
